# Proceeding of the International Digital Dentistry Society World Congress, Baden Baden 2019: Baden Baden, Germany. 3-6 October 2019

**DOI:** 10.1186/s12903-019-0954-7

**Published:** 2019-12-03

## The Digital Dentistry Society Poster Session- 3^rd^ October 2019, Kurhaus Baden Baden, 9am- 3pm

### Science

#### P01 Quantitative accuracy of cone-beam computerised tomography (CBCT) derived biomodels

##### Sohaib Shujaat, Eman Shaheen, Constantinus Politis, Reinhilde Jacobs

###### OMFS IMPATH Research Group, Department of Imaging and Pathology, Faculty of Medicine, University of Leuven and Oral & Maxillofacial Surgery, University Hospitals Leuven, Leuven, Belgium

####### **Correspondence:** Sohaib Shujaat (sohaib.shujaat@kuleuven.be)

**Abstract**

Background: Biomodeling in integration with digital imaging techniques such as intra-oral scanning and cone-beam computerized tomography (CBCT) is being commonly practiced in dental implantology for pre-operative treatment planning, clinical teaching and evaluating bone density and drilling techniques. The objective of following study was to assess the quantitative accuracy of CBCT-derived mandibular biomodels.

Methods: A CBCT dataset was segmented and mandible was isolated. Fifteen mandibular biomodels were fabricated utilizing multijet (MJ=4), digital light processing (DLP=4), stereolithography (SLA=2), fused deposition modeling (FDM=2), colorjet (CJ=2) and selective laser sintering (LS=1) based high-end commercial and in-house printers. Printed models were superimposed onto the original mandible for comparing surface accuracy. A part comparison analysis with color-coded map was carried out for evaluating the difference between each printed and original model surface.

Results**:** MJ technology showed least amount of error associated with both erupted and impacted teeth. Highest bony surface accuracy was achieved with SLA and LS based biomodels. Trabecular structures were most accurately printed with MJ printers. FDM technology had the highest overall absolute mean error associated with both teeth and bone. No significant differences were observed between each surface of original and printed models.

Conclusions: Overall biomodels were able to well replicate skeletal and dental anatomical surfaces. However, some models showed shortcomings in relation to depicting trabecular architecture which might influence bone density for drilling bone and placing dental implants.

#### P02 A new system for augmented reality composite dynamic vision of a surgical site for medical device guided placement

##### Marco Farronato^1^, Davide Farronato^2^

###### ^1^Department of Orthodontics, Fondazione IRCCS Ca’ Granda, Ospedale Maggiore Policlinico, University of Milan, via Francesco Sforza 35, 20122 Milano, MI, Italy; ^2^ School of Medicine and Surgery, University of Insubria, Via G. Piatti 10, 21100 Varese, Italy.

####### **Correspondence:** Marco Farronato (marcofarronato@msn.com)

**Abstract**

Background. On the base of the state of the art on the applications of augmented reality (AR) in dentistry from previous studies a new system was developed and *in vivo* tested with Institutional Review Board approval of the Ethics Committee of Policlinico of Milan (n. 421). The purpose of the new system is to provide a new light, portable hand-held smart device which provides a compound superimposed dynamic vision of the anatomical structures of the patient, radiologic exams and digitally positioned medical devices for AR-guided operations.

Methods. CBCT data and the .stl files of miniscrews (TADs), (Leone, Firenze, Italy), were imported in a dicom viewer, placed in the correct position and fixed, then exported to .stl format separately. The digital images were uploaded to a server and opened with a smart, light hand-held hardware. The hardware was placed in front of the patient placed in N.H.P and the new software was started. Superimposition was regulated with pinch gesture on the touch screen until correct positioning of the frontal teeth of the patient, the opacity of digital images were regulated with a function of the software. The superimposition was automatically stabilized by a function of the software using software development kit (SDK) engines. Tads were placed under the guide of the compound vision. Time measurements were taken on superimposition by different operators and the error was calculated with a digital caliper.

Results. The new system provided effectively a dynamic functional compound superimposition of anatomical structures and previously digitally placed medical devices. The error in the positioning of the TADs was calculated as < of 0.3 mm for both while time measurements of the superimposition session were less than 5 minutes for each session.

Conclusions. The new proposed system effectively provides a simple and light dynamic AR superimposition of digital data on a surgical site providing the clinician a compound vision of anatomical structures. The TADs were placed with AR assisted surgery with a acceptable error, further in vivo and in vitro studies are needed.

#### P03 Flat to Flat Vs Magic Five Cone Morse Connection regarding height/width ratio of peri-implant buccal soft tissues

##### Cristian Scognamiglio^1^, Stefano Ponti^2^, Veronica Campana^3^, Davide Farronato^4^

###### **Correspondence:** Cristian Scognamiglio (c.scognamiglio93@gmail.com)

####### ^1^DDs, Private Practitioner, Milan, Italy; ^2^DDs, Private Practitioner, Varese, Italy; ^3^Dentistry Student, University of Insubria, Varese, Italy; ^4^PhD, PD, AP, Assistant Professor, University of Insubria, Varse, Italy

**Abstract**

Purpose. Modern implantology has been focused on the peri-implant mucosa maturation patterns, with particular attention on the frontal area, aiming to obtain an optimal aesthetic result. The purpose of the following study is to analyze height/width proportion in the peri-implant buccal tissues between two different types of connection: Flat to Flat and Magic Five Cone Morse Connection (Anyridge®).

Methods. The present study analyzes 294 implants, the measurements were done with digital 3D technology at one year from the provisional crown placement. Every single implant was matched with its own Scan Body and was scanned with 3Shape D700, in order to obtain a tridimensional section of the model. Peri-implant measurements included the width of the peri-implant mucosa (HV), calculated from the implant connection to the buccal mucosa surface on a line perpendicular to the main implant axis. For the height of the peri-implant tissues (VV), we considered as well, the same geometrical line as the apical point of the measure to calculate the perpendicular distance to the most coronally gingiva point. All the recorded data were analyzed with Test Pearson 2-Tailed (95% Confidence Interval) by IBM® SPSS® Statistics Software. The study protocol was approved by the local Ethics Committee.

Results. The average ratio between height/width of the two different types of connection were compared. Flat to Flat Connection shows an average VV:HV ratio equal to 1:1,54. Meanwhile, Magic Five Cone Morse Connection has an average ratio of 1:1,18. This results are statistically significative at p ≤ 0,001 (Pearson 2-Tailed).

Conclusions. This study based on 294 implants approved Nozawa’s past observations on 14 cases: the average ratio of VV:HV in Flat to Flat connection is 1:1,54. Analyzing the new Magic Five Cone Morse Connection results, the average ratio VV:HV is 1:1,18, showing higher efficiency on biological integration tending to the natural tooth parameters investigated by Wennström: VV:HV = 1,5:1. The differences discovered between the two types of connections can be linked to the “Pumping Effect” described by Zipprich and these data refers to one year maturation period. Therefore, when a transverse load is applied on a Flat to Flat Connection, there is a tendency to create a micro gap between the implant and the prosthetic abutment interface. The presence of the gap induces micro movements that may work as a pump allowing bacterial endotoxins contamination, compromising the peri-implant soft tissue trophism.

#### P04 How to optimize tissue for the Coronally Advanced Flap. Interaction between emergence profile and tissue tropism

##### Luciano Laveglia^1^, Veronica Campana^2^, Marco Colombo^3^, Davide Farronato^4^

###### ^1^DentistryStudent, University of Insubria, Varese, Italy; ^2^DentistryStudent, University of Insubria, Varese, Italy; ^3^DDs, Private Practitioner, Milan, Italy; ^4^PhD, PD, AP, Assistant Professor, University of Insubria, Varse, Italy

####### **Correspondence:** Luciano Laveglia (l.laveglia@outlook.it)

**Abstract**

Background. Several operative protocols recommend the realisation of cervical restorations to restore CEJ in patients undergoing mucogingival plastic surgery.The purpose was to evaluate the response of gingival tissues following the emergency profile restoration with , in patients subsequently subjected to Mucogingival surgery.

Methods. Data were collected from 50 teeth, 47 of which were affected by vestibular recession and 3 without recession.The STL files for the 2 stone models made after the alginate imprint detected prior the conservative therapy (TO) and after at least 60 days (T1) were analyzed.The three dimensional matching of the 2 models was carried out by the GeomagicSoftware and subsequently, with Meshmixer, a series of ortho-shaped and parallel (cutter) planes were superimposed (0 + 0.5 mm, 0 + 1mm, 0 + 1.5 mm) in the Coronal and apical direction considering how to plan 0 the T0 buccal zenith of the analyzed element. The desired measurements were obtained using 3D Viewer visualization software, going to measure the distance between the cutter intersections with the models T0 and T1. The study was conducted in accordance with the fundamental principles of the Helsinki Declaration and was approved by the Ethics Committee at Insubria University.

Results. After the modification of the emergency profile, the thickness and height of the gingival tissue at the level of the free gingival margin has increased statistically significantly with a ratio of the 2 variables is equal to 1.183. Moreover, the increase in tooth thickness corresponds to an increase in the thickness of the gingival margin at the zenith according to a ratio of 1.8:1.Moreover, in subjects with Biotype 2 (festonate), a greater reduction in the recession was detected compared to those with biotype 1 (thin).

Conclusions. The realization of 5-class restorations with an increased emergence profile performed 2 months prior to the periodontal surgery shows clinical advantages.

#### P05 Implementation of Automated Digital Odontometry in Studies of Tooth Crown Morphology

##### Armen Gaboutchian^1^, Kristina Goryainova^2^, Samvel Apresyan^2^, Vladimir Knyaz^3,4^

###### ^1^Moscow State Medical-Stomatological University, Moscow, Russia; ^2^Peoples Friendship University of Russia, Moscow, Russia; ^3^State Research Institute of Aviation System, Moscow, Russia; ^4^Moscow Institute of Physics and Technology, Moscow, Russia

####### **Correspondence:** Kristina Goryainova (kristina@dda-russia.ru)

**Abstract**

Background. To propose a method for objective, robust and precise assessment of tooth crown (natural of artificially designed) morphology, where tooth crown can be taken as a whole or in its integral parts, being analysed through variability of measured or calculated parameters. Related matters dealing with basic interpretation of dental morphology, formalisation of measurements and orientating of measured teeth are presented as well.

Methods. Experimental part of the study has been carried out on a sequence of 3D images obtained from dental arch casts, experimentally prepared teeth on the same stone cast models and modelled (digitally and manually) artificial crowns. Digitally modelled crowns were assessed in their default morphology and after reshaping by technician. A significant source of studied samples relates to palaeoanthropological findings, which refer to various historical periods: Upper Palaeolithic, Early and Middle Bronze, Middle Ages and Modern Era. The studied images were obtained on dental and technical professional equipment (intraoral and laboratory scanners), custom designed photogrammetric scanners as well as on CBCT and micro-CT scanners. The scanning equipment, being chosen in accordance with the studied objects’ characteristics, provided high resolution digital images. Merged images of objects, obtained on various stages of the study or obtained on various equipment, were used as well. Measurements based on the proposed methods were carried out by means of software which provided, after 3D image loading, automated procedures of orientating, multidirectional sectioning modes, measurement landmark (point) detection, distance measurements, contour measurements, calculations, parameter presentation and analytical tools. Up to two hundred parameters are suggested in the current software version on each studied tooth section; the number of studied sections in the existing software version is limited by one hundred. The odontometric data includes linear, angular, contour parameters and coefficients referring to the whole crown or its parts.

Results. Automatically generated odontometric data, obtained on series of images: intact tooth – manually modelled crown – suggested “on default” digital crown shape – reshaped digitally modelled crown, was compared in reference to maximal vestibular-oral dimension. Assessments included estimation of value and localisation of measured areas on tooth crown.

Conclusions. Automated digital odontometry has demonstrated a potential for conducting measurements of tooth crowns. According to the obtained data, digital method of crown modelling can be considered more accurate and preferable in various terms of their usability.The work was performed with the support by Grant 17-29-04509 of Russian Foundation for Basic Research (RFBR).

#### P06 Analysis of the Height/Width ratio of peri-implant soft tissues in the different oral sectors

##### Stefano Ponti^1^, Cristian Scognamiglio^2^, Veronica Campana^3^, Davide Farronato^4^

###### ^1^DDs, Private Practitioner, Varese, Italy; ^2^DDs, Private Practitioner, Milan, Italy; ^3^Dentistry Student, University of Insubria, Varese, Italy; ^4^PhD, PD, AP, Assistant Professor, University of Insubria, Varse, Italy

####### **Correspondence:** Stefano Ponti (steponti@hotmail.it)

**Abstract**

Purpose. During the past years, frontal areas rehabilitation has been one of the most important topic of discussion in Implantology. The aim of a good anterior restoration, implant supported, is to achieve function and aesthetic result. The purpose of the following study is to analyze using 3D technology the height/width proportion in the peri-implant buccal tissues comparing different areas in the oral cavity.

Methods. The present study analyzes 294 implants, the measurements were done with digital 3D technology at one year from the provisional crown placement. Every single implant was matched with its own Scan Body and was scanned with 3Shape D700, in order to obtain a tridimensional section of the model. Peri-implant measurements included the width of the peri-implant mucosa (HV), calculated from the implant connection to the buccal mucosa surface on a line perpendicular to the main implant axis. For the height of the peri-implant tissues (VV), we considered as well, the same geometrical line as the apical point of the measure to calculate the perpendicular distance to the most coronally gengiva point. The Implants were split into four main groups on the base of their position in the oral cavity:

1. Anterior Maxilla
2. Anterior Mandible
3. Posterior Maxilla
4. Posterior Mandible

All the recorded data were analyzed with Test Pearson 2-Tailed (95% Confidence Interval) by IBM® SPSS® Statistics Software.

Results. The average height/width ratio in the different areas of the oral cavity shows a significative difference between the anterior sectors and the posterior one.

1. Anterior Maxilla 1:1.23
2. Anterior Mandible 1:1.24
3. Posterior Maxilla, 1:1.45
4. Posterior Mandible, 1:1.77

These results are statistically significative at p ≤ 0,001 (Pearson 2-Tailed).

Conclusions. This study based on 294 implants approves Nozawa’s past observations on 14 cases: the average ratio of VV:HV is overall 1:1.54. Analyzing the differences between the four areas, we can assert that in the aesthetic sectors there is a ratio that follows the height of peri-implant soft tissue; on the other hand in the posterior sectors the ratio is unfavorable to the tissue height. These results might be linked to two different factors: a greater thickness of posterior soft tissues and the role of Buccinator Muscle which during the impression phases is able to pull the vestibular fornix reducing its height. In addition we should not forget that the aesthetic areas requires a specific attention regarding transparency of the abutment caused by thin width of the soft tissues.

#### P07 Key role in impression accuracy: transfer types or implant placement angulation? An in vitro 3D evaluation

##### Veronica Campana^1^, Cristian Scognamiglio^2^, Giada Goffredo^3^, Davide Farronato^4^

###### ^1^Dentistry Student, University of Insubria, Varese, Italy; ^2^DDs, Private Practitioner, Milan, Italy; ^3^DDs, Private Practitioner, Milan, Italy; ^4^PhD, PD, AP, Assistant Professor, University of Insubria, Varse, Italy

####### **Correspondence:** Veronica Campana (v.campana@studenti.uninsubria.it)

**Abstract**

Background. Accurate impression making is a fundamental prerequisite such to achieve a passive fit between the implant and the prosthetic structures. The aim of this study is to evaluate if there are differences in the accuracy of the impression using three different types of transfer and to analyze their efficiency in comparative situations given by different angles of implant placement.

Methods. An acrylic resin master model with four implant analogues placed at 0, 15 and 35 degrees towards the horizontal plan was used. Twenty-seven polyvinyl siloxane impressions of the model were made using acrylic resin individual impression trays with the aim of evaluating three different types of transfer: a closed tray technique transfer, a classic open tray technique transfer and a telescopic open tray transfer. The impressions were poured with type IV die stone. After matching each analogue with a digital scan body, an STL file was achieved with the scanner “3SHAPE D2000”. An implant bar was projected from all the different STL files with “3 Shape Dental System” software. Each project is originated from each different impression in order to gain the implant position referred by the transfer under analysis. A comparison between this bars and the one projected on the master model was obtained with “Magic Materialise 13.0” software. Both a linear and angular measurement (heads in x, y and z axes) for every type of transfer, valuated in different angulation, was achieved. The collected data have been processed by the statistical software “IBM SPSS Statistics, Armonk, New York, United States” and analyzed with student’s T test and Kruskal-Wallis not parametric test.

Results. Even if the student’s T test revealed a statistical significance of the different linear and angular measurements in ∆x, ∆y, ∆z changing the transfer’s type, more restrictive tests have highlighted a not statistical significance related to the transfer’s type. However, when considering a variation of the implant positioning angle, the same tests revealed a statistically significant difference.

Conclusions. Considering the limitations of this study, it could be concluded that the type of transfer used is not a significant parameter rather than the implant positioning showed a significant key role in the precision success of impressions.

#### P08 Drilling protocols in guided surgery: an in vitro study

##### Nicolò Vercellini^1^, Veronica Campana^2^

###### ^1^Nicolò Vercellini DDS; Research Fellow, ITEB “Innovative Technology and Engineered Biomaterial” Research Centre, director Prof. Alberto Caprioglio. Department of Medicine and Surgery; University of Insubria, Varese, Italy; ^2^Dentistry Student, University of Insubria, Varese, Italy

####### **Correspondence:** Veronica Campana (veronica.campana93@gmail.com)

**Abstract**

Background. Demonstrate the better accuracy of a computer-guided surgery that follows a complete drilling protocol which includes all the steps in order to reach the whole depth of implant placement. The most apical point is where may occurs the largest angle of deviation from the projected one and where the drill may not be initially guided by the sleeve. As a conclusion, this in-vitro-study is meant to demonstrate the better precision of a real double-guided surgery both from the progressive osteotomies and from the guide of the entry point.

Methods. A 3D model was created, projecting the positioning of a Ø3.8 x 13mm Camlog Conelog implant and a teeth supported template for guided surgery, with incisal-level windows in order to verify its right positioning on the dental elements. Dental SG resin (Formlabs) is the printing material used. Two kind of preparations are compared:

- A complete sequence from this moment indicated as CIP (complete implant preparation)
- A reduced sequence, indicated as RIP (reduced implant preparation), which doesn’t include all the gradual steps of drilling preparation in length.

The osteotomies of the two sequences, CIP and RIP, are performed. The implants are positioned in the two models and subsequently, after the connection to a scan body, the model are acquired with an industrial certified scanner “3SHAPE D2000” (documented 5 micron accuracy ISO 12836; 4 × 5.0 MP cameras). Two STL files are obtained.

The STL files are upload in Magics Materialize software and both implant positions (obtained with CIP and RIP) are compared with the initial implant positioning project.

In this way it was possible to verify the real implant position obtained with the complete CIP sequence and with the reduced sequence RIP.

Results. The superiority of the complete protocol (CIP) on the reduced one (RIP) is demonstrated. Compared to the implant placement of the initial project the RIP protocol gave a distance of 0.7823 mm and an angle of 1.42 deg; Instead, with the CIP protocol the implant was placed at a distance of 0.5235 mm and at an angle of 0.56 deg, always referred to the initial project.

Conclusions. Within the limitations of this in-vitro study it can be concluded that, in order to achieve a greater accuracy in guided surgery, it is recommend a progressive drilling protocol that guarantees a guidance provided not only by the sleeve, but also by the progressive osteotomies.

#### P09 Implants placed at 35 degrees: is a telescopic transfer more reliable? An in vitro 3D evaluation

##### Veronica Campana^1^, Luciano Laveglia^2^, Marco Colombo^3^, Davide Farronato^4^

###### ^1^Dentistry Student, University of Insubria, Varese, Italy; ^2^Dentistry Student, University of Insubria, Varese, Italy; ^3^DDs, Private Practitioner, Milan, Italy; (4) PhD, PD, AP, Assistant Professor, University of Insubria, Varse, Italy

####### **Correspondence:** Veronica Campana (v.campana@studenti.uninsubria.it)

**Abstract**

Background. The aim of this study is to compare the accuracy of three different transfers and to analyze their impression’s accuracy in an high implant angulation at 35°. In addition to the classic transfers for the open tray and closed-tray impression techniques, it’s tested also a telescopic open tray transfer. This last one is characterized by an inner hexagon that can sweep inside the outer body such to eliminate any extension of the connection under the conical interface. Moreover this study is meant to verify the incidence of the relative angulations between the transfer as well as if a 35° angulation prepone a favorite choice depending on this angle. The null hypothesis to validate was that there would be no differences in 3D accuracy between 3 different transfers when tested at 35°.

Methods. A resin master model with 4 implant analogues placed at 0 and 35 degrees towards the horizontal plan was used. 27 polyvinyl siloxane impressions were made using acrylic resin individual impression trays with the aim of evaluating 3 different types of transfers. The impressions were poured with type IV stone. After matching each analogue with a digital scan body a STL file of the impressions was achieved with “3SHAPE D2000” scanner. An implant bar was projected from all the STL files with “3 Shape Dental System” software. These bars indicate the implant positions referred by the transfer under analysis. A comparison between this bars and the one projected on the master model was obtained with “Magic Materialise 13.0” software. Both a linear and angular measurement for every type of transfer, valuated in different angulation, was achieved.

Results. All the measurements collected have been processed by the statistical software “IBM SPSS Statistics”. The not parametric Jonckheere-Terpstra test was performed and astatistically significant difference between the transfer types at 35° inclination was found (p=0.023) when considering the angular displacement between them.

Conclusions. Within the limitations of this study it can be concluded that, even if the presence in the telescopic transfer of an inner hexagon that can sweep inside the outer body represents one more element of tolerance between the transfer body and the internal retractile hexagon, when tested at 35° it shows a greater precision and so the advantage to use a telescopic transfer in presence of tilted implants at high angulations seems the ideal solution.

#### P10 ROBOTA: From Digitalization to Automation & Data Analytics

##### Giorgio Castagno^1^, Veronica Campana^2^, Riccardo Castagno^3^

###### ^1^Doctor of Dental Surgery, Private Practitioner, Borgosesia, Italy; ^2^Dentistry Student, University of Insubria, Varese, Italy; ^3^Management Engineering Student, LIUC University, Castellanza, Italy

####### **Correspondence:** Giorgio Castagno (giorgiocastagno@yahoo.it)

**Abstract**

Background. Expose the project of an automated sterilization chain and the related advantages, such as the reduction of high cycle-times, of the elevate risk of contaminations and of the huge production of waste. These are enemies that make the daily process stressful. We are reorienting the sterilization chain into a manufacturing system in order to obtain jobs’ continuous flow. Through value stream mapping, a method for analyzing the current state of events that take place from the beginning of the process to its end, we in fact observed that a change in the sterilization process is needed.

Methods. The low value processes in dentistry operations are analyzed with “Lean and Six Sigma Dentistry”. An analysis of “Industry 4.0 system” that handle the sterilization is performed from the infected utensils’ carriage, with an Automated Guided Vehicle, to the disinfection, continuing to the wrapping process and storage using robots (Kr3 Agilus KUKA, Grugliasco, Italy). An integrated vision system scan which tools the system is processing: data are acquired during the entire process. ROBOTA can represent a powerful tool to optimize sterilization routine processes and to increase utensils fulfillment.

Results. ROBOTA can reduce the time spent to sterilize surgical instruments. It can works 24/24h so operators can dispose of a bigger amount of envelopes’ stock such to satisfy every demand. In this way it’s not necessary to buy same utensils multiples time. It’s also able to track the tools consumption so that the utensils’ lifespan can be exploited as maximum as possible. In addition, optimizing the whole process chain, less energy is required from the autoclave, whereas less heat is dissipated from the waiting time between one cycle to the next. The digital processes are automated, so manpower’s presence and human error is not only reduced, but also eliminated, and not only for patients, but also for operators. ROBOTA can change the medical world, eliminating risky jobs and creating a higher level of service. This is an impactful tool that ensure to patients that the risk of contamination is close to 0 and the service can be faster. We’re now able to connect ROBOTA with our agenda through Data Analytics: an algorithm examines data created from the system and draws info that can improve the way our Customer Management System program our daily clinical schedule.

Conclusions. ROBOTA can help to achieve the goal of reducing contamination risks and errors, ensuring at the same time higher level of sterilization.

#### P11 Jaw elevator muscles activity and pain on palpation with clear aligners. A prospective observational study

##### Alessandro Nota, Atanaz Darvizeh, Simona Tecco

###### **Correspondence:** Alessandro Nota (dr.alessandro.nota@gmail.com)

####### University Vita and Salute San Raffaele, Milan, Italy

**Abstract**

Background. It is not clear, from the available literature, whether the orthodontic treatment with clear aligners, which has a not negligible thickness between the dental arches, is accompanied by adaptive changes in the electrical activity of the jaw elevator muscles. The aim of this study was to report the jaw elevator muscles activity and their pain on palpation during the early stages of orthodontic dental alignment with clear aligners.

Methods. Surface electromyography (sEMG) and pain level on muscle palpation of masseter and anterior temporalis muscles, were recorded in a sample of 16 adult subjects (18-32 years old; mean 22.5 +/- 3.5 SD) undergoing to orthodontic treatment with clear aligners, Data were recorded before the treatment (T0), after 1 month of treatment (two clear aligners) (T1), and after 3 months of treatment (T2).

Results. No statistically significant differences in muscular pain were observed over the time in the whole sample. At T1, in the test group, the electrical activity in the masseter area at mandibular rest position showed a statistically significant reduction respect to the baseline values, but after 3 months (T2), data appeared similar to T0 (p=0.03 and p=0.02).

Conclusions. During the early stages of an orthodontic treatment with clear aligners, the subjects could experience an initial reduction of the basal electrical activity of jaw elevator muscles, which tends to disappear quickly. The behavior of the muscles during the orthodontic dental alignment deserves to be investigated in the future because preliminary data indicate a certain muscular adaptation to orthodontic movements, that could occur at least during the very early stages of treatment with aligners.

#### P12 Comparison of the accuracy of 3-dimensional printed dental models manufactured with different additive technologies

##### Török Gréta, Borbély Judit, Hermann Péter, Barbara Kispélyi

###### Department of Prosthodontics, Semmelweis, Budapest, Hungary

####### **Correspondence:** Török Gréta (drtorokgreta@gmail.com)

**Abstract**

Background. There are several different types of three-dimensional 3D printing technology that are appropriate for constructing dental models. In some cases, the cast has only a holder and carrier function during the whole digital workflow. Models with dies manufactured by additive technology can be used during the construction of extended fixed partial denture. The purpose of this in vitro study was to assess the accuracy (precision and trueness) of 3D printed dental models and models with removable dies manufactured with scan LED technology (SLT) and stereolithography (SLA) and PolyJet printers.

Methods. A digital upper jaw model serves as a reference. In the initial model, the 15 and 16 teeth are missing, the 11, 14 and 17 teeth are prepared with supragingival chamfer preparation for fixed prosthetic appliance. The 26 tooth was prepared for inlay. To standardize the measurements 16 markers (1 mm diameter) were created on the digital model. After designing the reference markers the model was sectioned to get a model with removable dies using 3Shape Model Builder program. The physical dental models were printed 3 types of 3D printers and printed 5 times with each printer. 1. SLA technology (FormLabs Form2 printer, Dental Model Resin, with layer thicknesses 50 μm). 2. SLT technique (MediTech D30 printer, FotoDent Model material, layer thickness 50 μm) 3. PolyJet technique (Objet Eden 350V 3D printer, VeroFlexWhite, layer thickness 14 μm). The 3D printed models and sectioned models were scanned using a high-resolution industrial 3D scanner (3D System) within two weeks after printing. The accuracy of 3D scanning is ± 0,05 mm. The scanned models were saved as stl format and imported into the Geomagic Verify software to analyse the precision and trueness of 3D printers. After the superimposition, the whole deviation and distances between predefined reference points were measured with a digital calliper. Measurements for the 3 types of printed models were compared with the initial digital model. The deviation of 50 μm has been defined as the allowed deviation interval in prosthetic aspect.

Results. The accuracy of the investigated Objet, Meditech D30 and FormLabs Form2 printers is within the allowed deviation interval.

Conclusions. Within the limitation of this study it was concluded that PolyJet, SLT and SLA technologies are able to fabricate a clinically acceptable physical model that is essential for construction of high-precision fixed prosthetic appliances.

#### P13 In vitro study on digital splint effect to the accuracy of digital dental implant impression

##### Gedrimiene Agne^1^, Rutkunas Vygandas^2^, Auskalnis Liudas^3^, Jegelevicius Darius^4,5^, Dirse Julius^6^, Bilius Vytautas^7^, Pletkus Justinas^8^

###### ^1^Phd Student, Department of Prosthodontics, Institute of Odontology, Faculty of Medicine, Vilnius University; “Prodentum” Private Practice. Vilnius, Lithuania; ^2^Associate Professor, Department of Prosthodontics, Institute of Odontology, Faculty of Medicine, Vilnius University; “Prodentum” Private Practice. Vilnius, Lithuania; ^3^Student, Institute of Odontology, Faculty of Medicine, Vilnius University. Vilnius, Lithuania; ^4^Head of Laboratory, Biomedical Engineering Institute, Kaunas University of Technology. Kaunas, Lithuania; ^5^Associate Professor, Department of Electronics Engineering, Kaunas University of Technology. Kaunas, Lithuania; ^6^Dentist, “Prodentum” Private Practice. Vilnius, Lithuania; ^7^Dentist, “Vilnius University Hospital Clinic of Žalgiris”. Vilnius, Lithuania; ^8^Dentist, Institute of Odontology, Faculty of Medicine, Vilnius University, Lithuania; “Prodentum” Private Practice. Vilnius, Lithuania

####### **Correspondence:** Pletkus Justinas (justinas.pletkus@gmail.com)

**Abstract**

Background. Since IOS devices can only capture part of the object at a time, images have to be stitched together to form a 3D object and therefore it is the source of possible errors of the scan. The aim of this in vitro study was to compare the trueness and precision of three different IOS scanning partially and fully edentulous models with 2 or 4 implants with attached scan bodies and digital splints.

Methods. Two types of maxilla models were printed with AsigaMax 3D printer. The first model was missing both premolars and molars on the right side, so Straumann BL dental implants were inserted instead first premolar (straight) and second molar (tilted 20°mesially). Four implants were inserted in the second edentulous model symmetrically at second incisors (straight) and first molar areas (tilted 20° mesially). Scan bodies were attached to the implants and models were scanned with Nikon Altera 10.7.6. coordinate measurement machine (CMM) to form a reference scan. DII was taken with a Primescan (version 5.0.1), CS 3600 (version 3.1.0), Trios3 (version 1.18.2.10) IOS ten times each (n=10) without digital splint. After that, tablets of hardened Fuji Plus cement was glued in edentulous areas to form digital splint and all models were scanned with three different IOS. Scanning data was exported in standard tessellation language format for analysis. All scans were aligned on the reference scan precisely by applying best-fit alignment procedure. Distance and angulation between scan bodies were measured aligning CAD models of scan bodies to the scanned surfaces of scan bodies.

Results. Trueness of distance and angle in Carestream partially edentulous models was 185 μm in the group with splint and 280 μm without one and 0.22° in the group with splint and 0.29° in the group without respectively. Precision of distance and angle measurements in the splint groups were 87 μm and 0.13°, in the groups without-202 μm and 0.25°. In fully edentulous models trueness of distance varied 53-106 μm in the groups with splint and 67-8μm in the groups without.

Trueness of Primescan in partially edentulous models with splints was 21μm and 0.16° in distance and angular measurements. Without splints-27μm and 0.21°. For fully edentulous models trueness and precision of distance and angle was better n groups with splint than without. Trueness of distance and angle of Trios3 in partially edentulous splinted models was 15 μm and 0.3°; 53 μm and 0.11° in unsplinted models respectively. For fully edentulous splinted models trueness of distance and angle varied 32-122μm and 0.19-0.51°, for unsplinted models-17-80μm and 0.1-0.45°.

Conclusions. Primescan showed the best results of trueness and precision of distance and angle measurements. Since digital splints improve the accuracy of DII, the impact of their forms and materials should be more researched.

#### P14 Effect of intraoral scanner, printer and digital analog system on accuracy of 3D printed models

##### L. Auskalnis^1^, D. Jegelevičius^2,3^, M. Akulauskas^2^, A. Gedrimiene^4^, T. Simonaitis^5^, R. Jurgaityte^5^, Vygandas Rutkunas^6^

###### ^1^Student, Institute of Odontology, Faculty of Medicine, Vilnius University, LITHUANIA; ^2^Engineer and Head of laboratory, Biomedical Engineering Institute, Kaunas University of Technology, LITHUANIA; ^3^Associate Professor, Department of Electronics Engineering, Kaunas University of Technology, LITHUANIA; ^4^PhD student, Department of Prosthodontics, Institute of Odontology, Faculty of Medicine, Vilnius University, LITHUANIA; ^5^Dental technician, UAB “ProDentum”, Vilnius, LITHUANIA; ^6^Associate Professor, Department of Prosthodontics, Institute of Odontology, Faculty of Medicine, Vilnius University, LITHUANIA

####### **Correspondence:** Vygandas Rutkunas (vygandasr@gmail.com)

**Abstract**

Background. Digital workflow for producing implant-supported restorations involves the usage of intraoral scanners (IOS). From IOS data 3D printed master model is often fabricated using the selected type of digital analogs. There is a lack of data regarding the effects of IOS, 3D printer, and digital analog type on the local and global accuracy of digital analog positions in 3D printed master model. Moreover, errors arising in each step/stage should be identified.

Methods. Two Straumann BLT 4.1 mm RC implants were inserted in the reference model (REF) left quadrant, in the location of second premolar with 0° angulation and second molar with 5° angulation. Three calibration spheres of 5 mm in diameter (±1 μm) where placed on the left quadrant at the model base. Scan bodies (3Shape) were attached to the implants and model was scanned with Nikon Altera 10.7.6. industrial scanner (REF-stl). REF model was scanned 10 times with E3 (3Shape) scanner for validation. Ten digital impressions were taken with Trios3 (3Shape) intraoral scanner (IOS-stl). The closest to the overall average of intraoral digital impressions STL file was selected for 3D printing. Asiga MAX and Next Dent 5100 3D printers were used to duplicate the reference model. Two types of digital implant analog systems were placed into the models: ELOS Print Model Analog and NT-trading DIM-ANALOG. In total 4 groups containing 10 quadrant 3D printed models were created. Later models were scanned with a validated E3 scanner (3D-print-stl). STL file superimpositions and measurements were performed using Geomagic Control X 2018 software. Distance, vertical shift, rotation and angulation measurements were made locally and globally.

Results. Validation procedure of E3 scanner showed the trueness of 26 μm and precision of 16.8 μm. Digital impression procedure with Trios 3 introduced 53 μm distance between the implants, 34.7 μm vertical shift, 0.283° angulation and 0.230° rotation errors locally. Comparison of REF_stl and 3D_print_stl files showed most accurate Asiga MAX and Elos PMA analog combination: 37.2 μm distance between the implants, 39.7 μm vertical shift, 0.212° angulation and 0.769° rotation errors locally. Majority of detected differences were statistically significant (p<0.05).

Conclusions. Asiga MAX 3D printer performed more accurately than NextDent 5100. ELOS Print Model Analog showed most accurate result both locally and globally than NT trading. Implant angulation of 5° provides more accurate analog position in the master model than 0° angulation. Intraoral scanning had significant influence in overall error propogation. Further studies are needed to evaluate other factors.

#### P15 Assessment of distortion caused by stitching during full arch intraoral scanning

##### János Vág, Evelin Kövér, Ákos Mikolicz, Zsolt Nagy

###### Department of Conservative Dentistry, Semmelweis University, Budapest, Hungary

####### **Correspondence:** János Vág (drvagjanos@gmail.com)

**Abstract**

Background. The field of application of today’s intraoral scanners is extending from making single tooth restorations towards full arch rehabilitations. Scanning technique (scan pattern) are suggested to influence the scanning accuracy especially in case of full arch. The aim of this study was to measure distortion of 3D models created with full arch digital impressions by a novel method and compare with two existing methods.

Methods. Maxillary and mandibular models were captured with an intraoral scanner using four different scan patterns. Accuracy and distortion were assessed by comparing master scans to the intraoral scans using the following three methods: 1: Mean surface deviation was measured after complete arch superimposition. 2: 28 points were selected identically on the experimental and on the master reference models. Deviation between identical points was assessed after superimposition over the complete arch. 3: (Novel technique) The same 28 points were compared after superimposition limited to the scanning origin.

Results. Significant differences were found between the three different methods regardless of the arch and pattern. The overall mean deviation between identical points when models were aligned at the scanning origin was the highest and the mean deviation between the non-identical values was the lowest. The new method revealed local, tooth-wise differences between scan pattern as well as pattern of error increasing with distance from scanning origin.

Conclusions. The new method better detects the cumulative deviation of stitching error in complete arch intraoral scans, and is suitable to investigate the effect of scanning pattern in a very sensitive manner.

#### P16 Comparison of distortion of seven intraoral scanners caused by stitching mechanism

##### Zsolt Nagy^1^, János Vág^1^, Anthony Mennito^2^, Walter Renne^2^

###### ^1^Department of Conservative Dentistry, Semmelweis University, Budapest, Hungary; ^2^Department of Oral Rehabilitation, Medical University of South Carolina College of Dental Medicine, Charleston, South Carolina

####### **Correspondence:** Zsolt Nagy (nagyzsolt.dent@gmail.com)

**Abstract**

Background. Usually full-arch accuracy of intraoral scanners (IOS) are measured by average deviation and does not taking into consideration the origin of scanning. However, during scanning, deviation is accumulating from the scanning origin till the last tooth on the arch. The aim of this work was to compare tooth wise kinetics of deviation of seven different IOS, all starting from the same scanning origin utilizing various hardware and software techniques.

Methods. Test digital models were produced by IOS of 7 different types on a human cadaver maxilla. All scans were started on occlusal surface of tooth 27 and ended up at tooth 17. Superimposition of test models with a master model were done in GOM Inspect software using a local best fit algorithm localized at the scanning origin. Deviation was measured between identical points and accumulated deviation (AD) was calculated at tooth 17. Deviation was also calculated separately on three axis, mesio-distal, bucco-lingual and apico-coronal. Data was statistically analyzed by generalized linear mixed model.

Results. The average full-arch deviation was 365±130 for CS, 535±116 for Element1, 247±27 for Element2, 320±48 for Emerald, 174±16 for Omnicam, 906±122 for Planscan, 156±25 for Trios3. AD was 524±92 for CS, 521±102 for Element1, 321±23 for Element2, 480±49 for Emerald, 408±21 for Omnicam, 1697±64 for Planscan, 235±18 for Trios3. These were not statistically different from each other except AD of Planscan which was significantly higher than all others. Deviation on apico-coronal axis at tooth 17 was higher than deviation measured on mesio-distal or bucco-lingual axis for Element2, Emerald, Omnicam, Planscan except CS, Element1 and Trios3. CS and Element1 had the highest coefficient of variation (56% and 79%) which might mask the difference in accuracy results.

Conclusions. Further away from scanning origin higher deviation values for all IOS were found and with most scanners, the deviation at apico-coronal axis related to the occlusal view was the highest. This suggests that proper selection of the tooth and the surface of origin may help to reduce deviation. The accuracy was improved considerably by manufacturers at the new generation of the same brand.

#### P17 Optical Properties of Monolithic CAD/CAM Zirconia Reinforced Lithium-Silicate Crowns

##### Alexandra Czigola^1^, Zoltan Imre-Kovacs^2^, Peter Hermann^1^, Judit Borbely^1^

###### ^1^Department of Prosthodontics, Faculty of Dentistry, Semmelweis University - Hungary – Budapest; ^2^Department of General Dental Preclinical Practice, Faculty of Dentistry, Semmelweis University - Hungary - Budapest

####### **Correspondence:** Alexandra Czigola (czigola.alexandra@dent.semmelweis-univ.hu)

**Abstract**

Background. All‐ceramic systems and CAD/CAM technics allow dentists to make monolithic restorations with reduced wall thickness. Glass‐ceramic restorations are in the focus of interest. When the right color block of glass-ceramic material is selected for the restoration it must be taken into consideration, that the final esthetics are influenced by underlying tooth color due to their translucency. The aim of this in vitro study was to determine how substrate colors, ceramic thickness and translucency, cement shades effect the final shade of CAD/CAM monolithic zirconia reinforced lithium-silicate (VITA Suprinity) crowns.

Methods. According to our previously published study1premolar teeth (14) were prepared on a study model for 1.0 and 1.5-mm thick full ceramic monolithic crowns. Intraoral scanner was used (Trios, 3Shape) to create digital impressions. CAD/CAM technics were used to design (Dental Designer) and make crowns with identical shape and size. Shade A1 crowns were milled (Everest, Kavo) from HT (high translucency) and T (translucent) Vita Suprinity (Vita Zahnfabrik) zirconia reinforced lithium-silicate blocks. 9 substrates were made of different color and materials (6 of Vita Simulate composite material and zirconia, Co-Cr, gold-colored alloy) to imitate substrate effects on the final shade. Three different try-in pastes were used to simulate the color effect of cements (Variolink Esthetic Try-In paste, Ivoclar). Shade measurement was done 3 times for each crown by spectrophotometer (Vita, Easyshade Advance) and averages were compared to a reference crown (A1, 1.5mm, T, 2M3S abutment, neutral try-in paste). ΔE00was calculated (CIEDE 2000 formula).

Results. All of the examined parameters influenced ΔE00of the CAD/CAM monolithic crowns. The weakest effect was exerted by the color of the try-in paste. When ∆E value smaller than 1.8 is regarded as a clinically not acceptable color change2, out of 108 measured combinations none of the HT and 19 of T crowns were below this threshold.

Conclusions. Before choosing the ceramic block translucency and restoration wall thickness we should consider the underlying abutment color, but final color was just barely affected by luting cement shade in case of 1.0 and 1.5 mm thick ceramic crowns.

### Clinics

#### P18 Post-Extractive Implant Placement in Upper Molar region: Implant Preparation Guided by Dental Roots

##### Christian Monti (monti.christian@libero.it)

###### Lake Como Institute, Como (ITA); Dental Student, University of Varese, Italy

**Abstract**

Background. The correct positioning of post-extractive implants in the upper posterior area is always a challenge, especially because of the maxillary sinus presence.The aim is therefore to propose, in selected cases, after a careful CT analysis, to use the roots themselves as an insertion-guide in order to obtain a clinical success.

Methods. A 45-years-old woman presented an important vestibular recession and a palatal fracture of element 16, requiring a morpho-functional restoration through implant-prosthetic rehabilitation. A CBCT was performed to evaluate the root trunk anatomy, the presence of inter-radicular bone and the maxillary sinus physiology. The 3D images showed divergent roots with a well-represented bone septum.In order to avoid drill off-setting through axial vibration, because not properly supported during the insertion, it was decided to prepare the implant site before extracting the roots: the tooth was uncrowned and the roots, thanks to their density and inclination, forming an effective guide for the correct three-dimensional osteotomy. The roots were then atraumatically removed, and the implant inserted.To avoid any buccal-bone resorption, a L-PRF Collagen Block was introduced in the peri-implant gap.

Results. A correct osseo-integration of the implant was documented, as well as an excellent response of the peri-implant tissues in the follow-up carried out in the two years succeeding the placement. A gingival re-modeling was achieved with the disappearance of the vestibular recession, because of the wound being allowed to heal by secondary intention and using the L-PRF membranes as a tissue healing boost.

Conclusions. The illustrated procedure remains a valid alternative, although not a substitute, of traditional surgical techniques which are already fully described in literature. This particular site preparation has allowed the obtainment of a perfect implant axis, a predictable implant positioning and osteo-integration, favoring the success of the implant-prosthetic rehabilitation.

#### P19 Fractured Premolar Replacement with Post-Extraction Implant and Immediate Loading with Guided Gingival Recession Regeneration - Fully Digital in Office

##### Fabrizio Pedrinis^1^, Jessica Dana^2^

###### ^1^Dental Studio Digitale – Via Pelli 13B Lugano (CH); ^2^Dental Student, University of Varese, Italy

####### **Correspondence:** Fabrizio Pedrinis (fabrizio@pedrinis.ch)

**Abstract**

Background. The replacement of the first upper premolar of a patient who showed up with a root fracture, with a post-extraction implant; delivering also a temporary crown in the same session, which aims a spontaneous gingival regeneration of the pre-existing buccal recession, for an additional aesthetic rehabilitation.

Methods. The CBCT confirmed the radicular fracture and showed a bone structure with a conserved vestibular theca that allowed the insertion of an implant right after the extraction. This 3D radiological exam was imported into the RealGuide (3Diemme) implant design software together with the dental arches scan obtained with the Omnicam intraoral camera (Sirona). The ideal positioning of the fixture was planned with the same RealGuide software, as well as the surgical guide which was designed and printed in office with a Form2 printer (Formlabs). The extraction was performed in an atraumatic way and a 4 x 13 mm Megagen implant was placed with the help of the surgical guide. The ISQ value was 78, which confirmed the good primary stability. In the same session a temporary PMMA crown was formed with the Cerec Chairside method (Sirona). Particular attention was paid to the creation of an S-shaped emergency profile in order to promote gingival regeneration.

Results. The implant was well osseo-integrated and after 3 months it was possible to observe a great gingival regeneration, which is always a prerequisite for a stable, aesthetic and functional result.

Conclusions. The fully-digital procedure performed entirely in office allowed the replacement of the fractured tooth within 2 hours. Thanks to the three-dimensional positioning guided according faithfully to the planification, and because of an accurate emergence profile design, a spontaneous gingival regeneration without any connective grafts was also obtained.

#### P20 Full Mouth Restoration with Immediate Loading

##### Alessandro Perucchi^1^, Christian Monti^2^, Jessica Dana^3^, Marco Del Prete^4^

###### ^1^Studio Odontoiatrico Dr.Alessandro Perucchi – Mendrisio (CH); ^2^Lake Como Institute, Como, Italy; ^3^Dental Student, University of Varese, Italy; ^4^Private Practice, Lugano, Switzerland

####### **Correspondence:** Alessandro Perucchi (alessandroperucchi@sunrise.ch)

**Abstract**

Background. To rehabilitate both jaws of a patient in bad oral conditions is already a challenge in itself. It was intentional to try a motivational training in order to obtain the perfect prerequisites for an implant-prosthetic rehabilitation with a digital workflow.Methods. A woman showed up with a poor oral hygiene, without been going to any dentist for 6 years. She went through a depression and was hospitalized for a year. Hence her desire to fix her teeth in a definitive way, in order to smile again.All the periodontal upper teeth were firstly extracted, and a removable prosthesis was delivered. Short after that her oral situation went really better, that’s why it was decided to subsequently rehabilitate the mandibular arch with a Toronto prosthesis immediate loaded on 6 post-extractive Megagen implants, all digitally planned.Considering that her oral hygiene was definitively ameliorated, it was chosen to deliver also in the upper jaw a fixed prosthetic solution immediately loaded on six BLX implants. The vertical dimension was reproduced with a digital wax up, and on the six BLX implants were screwed six SRA (screw retained abutment).

Results. The patient has shown to have definitively improved her oral hygiene, and for this reason there are all the good conditions for a long-term maintenance of the implants. After a year she is very glad, satisfied and has finally returned to smile without being ashamed of her appearance.

Conclusions. With the digital workflow it has been possible to deliver on both jaws an immediate prosthesis in a very short time. The result is extremely satisfying, since it was not only a technical and clinical challenge, but also a matter of caring this lady on a path of personal growth and reconquering her self-esteem.

#### P21 Development of an evidence-based application for dental grinding evaluation and sleep bruxism monitoring

##### Patricia Tersi, Cristiane Macedo

###### Department of Evidence-Based Medicine, Paulista Medical School, Federal University of São Paulo, São Paulo, Brazil.

####### **Correspondence:** Patricia Tersi (drapatriciatersi@gmail.com)

**Abstract**

Background. There are issues not clarified in the sleep bruxism (SB) field, some of them are associated to the present restrictions related to validity, sensitivity, specificity and poor correspondence between instrumental and non-instrumental diagnostic approaches. The current challenge is to establish more accurate, applicable, affordable and accessible approaches.

Aim: The aim of this study is to present and recommend the use of an innovative and technological smartphone-based approach for SB assessment. It is an evidence-based toll for tooth grinding evaluation and long-term sleep bruxism monitoring, enabling systematic data collection from SB self-report (I), clinical inspection (II) and tooth wear pattern (III).

Methods. The application uses principles of Artificial Intelligence (AI), bruxism consensus grade system statement and Ecological Momentary Assessment (EMA) to collect data from non-instrumental approaches with literature supported questions about self-report and clinical inspections. The image processing algorithms developed measure data from tooth wear pattern and its possible association with symptoms, comorbidities and risk factors.

Results. The findings of this study resulted in a smartphone-based approach for multifactorial SB assessment. With cloud storage, the data from this app can be used for clinical and research purposes.

Conclusions. This APP is an applicable and accessible approach for SB assessment, with algorithms that can contribute to a better understanding of the relationships between clinical assessment, self-report and tooth wear measurements.

#### P22 From intraoral scan to CAD-CAM no-prep partial veneers: a case report

##### Veronica Campana^1^, Giada Goffredo^2^, Luciano Laveglia^3^, Stefano Ponti^4^

###### ^1^Dentistry Student, University of Insubria, Varese, Italy; ^2^DDs, Private Practitioner, Varese, Italy; ^3^Dentistry Student, University of Insubria, Varese, Italy; ^4^DDs, Private Practitioner, Varese, Italy

####### **Correspondence:** Veronica Campana (v.campana@studenti.uninsubria.it)

**Abstract**

Background. To present the esthetic and orthodontic outcome of multiple lithium disilicate ceramic partial veneers, produced through a full digital process that includes an IOS impression and the computer-aided design/manufacturing phases. In this case report the advantages of this digital technique are presented.

Methods. A digital scan was taken with 3Shape TRIOS 3 Intraoral Scanner (1). Data were sent to the digital laboratory service where the veneers were designed using CAD/CAM design software (3shape dental system) and milled (2) from low translucency IPS e.max lithium disilicate (Vita A3) blocks (Ivoclar-Vivadent, Amherst, NY, USA) with a four axes milling machine (Vhf N4). A minimum thickness of 0.3 mm for the veneer and of 0.1 for the margins were set. A virtual marginal design was performed (3), checking with a 2D cross section the accuracy of the positioning of the veneer closing margin. The direction of insertion of the veneer was also designed and the occlusion was controlled through a virtual articulator. Veneers were then verified intraorally using a translucent try-in paste (Multilink Automix Try In, Ivoclar-Vivadent). All internal surfaces of the veneers were etched with 5% hydrofluoric acid for 25 seconds and silanated (Monobond Plus, Ivoclar-Vivadent). After conditioning the teeth surfaces with a primer (Multilink primer base/catalyst Ivoclar-Vivadent) the laminate veneers were cemented using a translucent light-cure resin cement (Multilink Automix Ivoclar-Vivadent). An occlusal check was performed, revealing the absence of interfering contact points both in protrusive and laterality. The study was conducted in accordance with the fundamental principles of the Helsinki Declaration and was approved by the Ethics Committee at Insubria University.

Results. The adaption and cementation to the teeth surfaces was clinically easy due to a well-designed planning phase that included not only the selection of the margin but also the detection of the axis of insertion: when this axis was involved in an undercut area, the software reports the error such to modify the design of the veneer. The aesthetic outcome of the lithium disilicate veneers was natural-looking and conservative, while providing high optical properties.

Conclusions. No-prep adhesive restorations are an excellent rehabilitative option for situations in which the dental elements are healthy and can be modified exclusively by an additive plane of treatment, for example if there is a need to compensate the limitations of an orthodontic treatment. The digital workflow allowed the fabrication of satisfying clinical results in terms of marginal fit, shape and aesthetics.

### Science

#### P23 Application of intraoral scanner to identify monozygotic twins

##### Botond Simon^1^, János Vág^2^, Ádám D. Tárnoki^3^, Dávid L. Tárnoki^3^

###### ^1^SCRUNCH Ltd., Budapest, Hungary; ^2^Department of Conservative Dentistry, Semmelweis University, Budapest, Hungary; ^3^Department of Radiology, Semmelweis University, Budapest, Hungary

####### **Correspondence:** Botond Simon (dr.simon.botond@gmail.com)

**Abstract**

Background. Current methods for identify the monozygotic (MZ) twinning are either invasive such as blood sample taking or expensive as DNA sequencing. Palatal rugae has been suggested previously as a marker of human identity. The aim of this study was to evaluate the 3D digital pattern of the palatum and rugae acquired by intraoral scanner in order to distinguish identical monozygotic twins from one another and to differentiate MZ from dizygotic twins (DZ).

Methods. Palatal area of 36 participants (MZ: 25; DZ: 11) was scanned three times with an intraoral scanner. The age of twins was between 18 and 60 years. Ethical approval was granted on July 26, 2018 by the Hungarian authority called Committee of the Health Registration and Training Center (approval number: 36699-2/2018/EKU). From the scanned data STL file was created and exported into GOM Inspect® inspection software. Each scan within a twin pair was superimposed to each other. The average deviation between scans of the same subject (reproducibility) and between scans of two subjects within a twin pair (intertwin comparison) were calculated with 95% confidence interval.

Results. The mean reproducibility of the palatal scan was 33 μm (31-36 μm). The intertwin deviation of MZ was 361 μm (324-402 μm) which was significantly (p<0.001) higher than the reproducibility values. The deviation of dizygotic twin pairs was significantly higher (+642 μm, 491-838 μm, p<0.001) than the deviation of monozygotic pairs.

Conclusions. MZ twins can be differentiated from each other accurately by palatal morphology obtained by intraoral scanner. This method may differentiate MZ and DZ twins as well but it requires further study. The alignment method is capable to identify monozygotic twin pairs. This could be a great advantage in forensic science and in twin studies.

### Clinics

#### P24 The facilitated esthetic orthodontic treatment with clear aligners and minimally invasive corticotomy: the importance of digital planning

##### Simona Tecco (simtecc@gmail.com)

###### San Raffaele University, Milan, Italy

**Abstract**

Backround. During the last decades, accelerating orthodontic tooth movement, has become a topical issue and there have been many attempts to shorten treatment duration. Among the different techniques described in the literature, the corticotomy seem to be an effective and safe mean. Corticotomy is an intentional injury to the cortical bone able to accelerate orthodontic tooth movement and dramatically reduce treatment times because it leads to a biological stage called regional acceleratory phenomenon (RAP) characterized an intensified osteoclastic activity, resulting in osteopenia and increased bone modeling. Nevertheless recently, the piezocision technique was introduced, performed under local anesthesia through a tunnel approach overcoming most of the disadvantages of traditional corticotomy. Simultaneously, orthodontic treatment with removable clear aligners has become an increasingly common treatment choice because of adult patients that desire aesthetic and comfortable alternatives to conventional fixed appliances. Being aesthetics and treatment duration the principal adult patients expectations, the aim is to illustrate the planning of a combined approach with piezocision corticotomy and clear aligners orthodontic treatment.

Methods. Two case reports are described, an open-bite and a closure of extraction spaces. After performing polyvinylsiloxane or digital impressions of the dental arches and sending it to the manufacturer a proper aligners orthodontic planning using the digital software (ClinCheck, Align Technology, Santa Clara, CA, USA) the orthodontist identified the time window in which the most difficult orthodontic movements had to be performed. The surgical procedure was planned and executed as starting point of this period in which each aligner will be used for 4 days rather than 15 days for a total time of 4 months. A careful planning of the ideal attachments for correctly expressing the aligner execution of these movements was necessary.

Results. Clinical results obtained were satisfactory results, as seen in figure 1 and figure 2 for the openbite and the space closure, respectively.

Conclusions. In order to underwent to accelerated esthetic orthodontic treatment (AEOT) applying a corticotomy surgical technique to orthodontic clear aligners treatment (Invisalign, Align Technology, Santa Clara, CA, USA), each subject should be carefully evaluated. A cone-beam computed tomography should be added to the standard orthodontic examination (anamnesis, extraoral and intraoral examination and photographs, dental casts, ortopantomography, cephalometry). The primary objective of this combination should be to facilitate difficult and less predictable orthodontic movement in addition to reduce the treatment duration, simultaneously, the long term risk of relapse should be reduced.

#### P25 Implant open-flap and flapless placement using dynamic navigation: a pilot controlled clinical trial

##### Gerardo Pellegrino (gerardo.pellegrino2@unibo.it)

###### University of Bologna, Italy

**Abstract**

Background. Dynamic navigation is a computer-guided technique offering different advantages with respect to conventional free-hand implant insertion. Some of these are an accurate and prosthetic-based implant placement, the surgical time reduction and safe use of a less invasive surgical procedure, like the flapless technique. This technique allows a higher patients comfort but is more challenging and is usually associated with less accurate placement of the fixture with respect to an open-flap method. The aim of this controlled clinical trial is to investigate the accuracy of implant placement comparing open-flap and flapless technique using a dynamic navigation system.

Methods.12 patients were consecutively recruited and allocated to the flapless or open-flap group depending on soft tissue parameters. Implant position was planned on the preoperative CBCT according to the prosthetic project using dedicated software. A total of 20 implants were placed: 11 with a flapless technique and 9 with the conventional open-flap method. The surgery was performed with the navigation system according to the virtual planning. The deviation between the real implant position obtained from the post-operative cone-beam computed tomography and the planned one was measured. Data were statistically analyzed using a Mann-Whitney U test.

Results. The errors of implant placement using a flapless approach were 0.88 ± 0.50 mm (range 0.46-1.84 mm) at the insertion point and 1.09 ± 0.62 mm (range 0.48-2.28 mm) at the apical point. The depth error was 0.37 ± 0.29 mm (range 0.03-0.86 mm) and the angular deviation was 4.78 ± 2.58° (range 1-9.7°). Using a conventional open-flap technique, the errors were 1.06± 0. 48 mm (range 0.45-2.21 mm), 1.40 ± 0.58 mm (range 0.59-2.21 mm), 0.45 ± 0.37 mm (range 0.18-1.41 mm), and 7.13 ± 4.76° (range 1.4-15.9°), respectively. The values of the two groups were not significantly different (p = 0.43, p = 0.27, p = 0.59,p=0.20). No difference was found between the conventional open-flap and flapless approaches in terms of the accuracy of implant placement.

Conclusions. Therefore, the use of a navigation system can overcome the drawback of a “blind” technique and ensure a high level of accuracy.

#### P26 Conventional and tilted implants placement using dynamic navigation: a case series

##### Gerardo Pellegrino (gerardo.pellegrino2@unibo.it)

###### University of Bologna, Italy

**Abstract**

Background. Tilted implants are used instead of bone regenerative techniques, allowing a more conservative approach. Dynamic navigation is a computer-guided technique allowing accurate implant placement and safe use of minimally invasive surgical procedures, like flapless and tilted implant insertion. The aim of this study is to report a series of cases in which implant placement was performed using a dynamic navigation system combining tilted approach and flapless technique. The secondary objective was to assess implants placement accuracy.

Methods. Three patients needing implant-prosthetic rehabilitation in the maxilla and having atrophy of the posterior area are treated (Ethics Committee approval number 104/2018/DISP/AUSLBO). For each patient, two implants (BTK, Biotec Srl, Italy) are planned, one of these tilted engaging the available alveolar bone and avoiding the maxillary sinus. The surgeries were performed with a dynamic navigation system (ImplaNav, BresMedical, Sydney, Australia) and all the implants were placed using a flapless approach. The implants were exposed after three months. The accuracy of implant placement was assessed measuring the deviations between the positioned and the planned implants comparing pre- and post-operative CBCT.

Results. No complications occurred and both tilted and conventional implants reached the osseointegration. The mean deviations of implants placed with a conventional technique were 0.93 ± 0.61 mm at the insertion point, 1.27 ± 0.78 mm at the apical point and 0.42 ± 0.29 mm in depth. The angular deviation was 4.53 ± 2.44°. The mean deviations of tilted implants were 1.03 ± 0.64 mm at the insertion point, 1.39 ± 0.81 mm at the apical point and 0.43 ± 0.24 mm in depth. The angular deviation was 4.62 ± 2.94°.

Conclusions. The use of dynamic navigation can be considered a reliable and safe technique in implantology. It allows the placement of implants using a conservative and safe approach even though more challenging procedures are performed. The accuracy values seem to be similar using a tilted or a conventional technique of implant placement.

#### P27 Augmented reality for dynamic navigation displaying: a report of two cases

##### Gerardo Pellegrino (gerardo.pellegrino2@unibo.it)

###### University of Bologna, Italy

**Abstract**

Background. Dynamic navigation is a computer-guided surgery technique used in dental implantology. The employment of navigation systems offers different advantages, as accurate placement of the fixtures. Many authors reported good results in terms of implant placement accuracy using this method. A drawback of this technique is the need to turn away from the surgical field to look at the monitor of the navigation system. Augmented reality (AR) is a technology employed in different surgical fields, as neurosurgery, vascular surgery, and maxillofacial surgery. Nevertheless, only few dentistry studies are available to date. The aim of this study was to present two clinical cases of navigated implant surgery associated with the use of augmented reality and to report the accuracy of implant placement using both the devices.

Methods. Two patients needing implant-supported rehabilitation in the upper premolar area were treated. Implant position was planned on the preoperative CBCT according to the prosthetic project using dedicated software. For each patient, an implant (WinSix, Ancona, Italy; Straumann, Switzerland) was placed by the same surgeon using a dynamic navigation system (ImplaNav, BresMedical, Sydney, Australia) and AR glasses (Fifthingenium, Milan, Italy).

Results. The navigation system allowed the operator to see the implant planning and to follow drills and implant position on the CBCT of the patient. With the association of the augmented reality device, the navigation screen was directly displayed near the surgical field. Both the implants were placed with a flapless technique. The accuracy of implant placement was evaluated measuring into CBCTs the deviation between real and planned positions of the implants. The deviation values for the first implant were 0.53 mm at the entry point and 0.50 mm at the apical point; for the second implant were 0.46 mm at the entry point and 0.48 mm at the apical point. The angular deviations were respectively 3.05° and 2.19°.

Conclusions. The use of AR glasses allowed the surgeon to have at the same time both the view of the surgical field and the position of the virtual drills without the need to turn away from the working position. The use of augmented reality applied to dynamic navigation allowed a reduced operating time and led to accurate values of implant placement. The association of these two technologies seems to be promising in dental implantology. More studies are needed to confirm these results.

#### P28 Esthetic performance of different CAD/CAM all-ceramic implant-supported crowns via digital workflows in the esthetic zone: a case report

##### Krisztina Mikulas^1^, Tamás Chikány^2^, Peter Hermann^1^

###### ^1^Department of Prosthodontics Semmelweis University, Maxdental - Hungary – Budapest; ^2^Intellident - Hungary – Budapest

####### **Correspondence:** Krisztina Mikulas (dr.mikulas@gmail.com, mikulas.krisztina@dent.semmelweis-univ.hu)

**Abstract**

Background. Successful esthetic outcome in implant rehabilitation can be resulted through accurate implant placement, management of supraimplant soft tissues and appropriate biomaterials. Provisional restorations (PR) have a decisive role in soft‐tissue contouring and preservation during maturation period after implant placement. Correct 3D capturing of the supraimplant emergence profile of PR allow predictable definitive restorations. This case report follows a recently known indirect scanning technique recommended in case of rapidly collapsed supraimplant mucosa and based on production of three different anterior all-ceramic implant-supported crowns via complete or semi digital workflow.

Methods. At baseline a 49 year old female patient with fracture of tooth 22 was treated. Immediate implant placement (BL, Straumann AG) was performed in a correct palatal and apico-coronal position, buccal gap was grafted with xenograft (Creos, Nobel Biocare AG). Immediate provisionalisation was carried out chairside. After healing period and creating harmonious supraimplant emergence profile we performed intraoral digital impression (CARES Intraoral Scanner) generating standard triangulation language files (STL) based on three digital impressions. The merged file of STL1, 2, 3 was used to design CAD/CAM abutments made of Y-TZP (Straumann CARES), single crowns and then a 3D stereolithographic model was printed for the semi-digital way. Two different CAD/CAM abutments were designed and milled for three different all-ceramic restorations. The outcome variables were the esthetic outcomes of the three all-ceramic crowns. Firstly a cement-retained monolithic zirconia crown on individualized CAD/CAM abutment was fabricated via complete digital workflow without any physical model. Next cement-retained pressed ceramic crown (IPS e.max CAD HT) was performed. Finally a one-piece screw-retained single crown based on CAD/CAM abutment veneered with hand buildup technique was delivered, which was finally inserted after the esthetic evaluation.

Results. The esthetic outcomes (PES and WES values) were pleasing overall for the three all-ceramic restorations.

Conclusions. Ideal shaping of provisional at immediate implant placement helps stabilize the bone graft material within the blood clot during healing and develop the desired emergence profile. The individualized zirconia abutment may provide an ideal surface for soft tissue adhesion with less plaque accumulation. Both complete digital and semi digital prosthetic pathways resulted in appropriate clinical performance in the esthetic zone and simplified the clinical procedures.

Written, informed consent for publication was obtained from the patient [or parent/guardian for patients under 16]

#### P29 Digital workflow to perform esthetic and functional rehabilitation of oligodontia with occlusal veneers and implant supported crowns, case report

##### Judit Borbely^1^, Peter Hermann^1^, Peter Windisch^2^, Szandra Körmendi^1^

###### ^1^Department of Prosthodontics Semmelweis University, Hungary – Budapest; ^2^Department of Periodontics Semmelweis University, Hungary – Budapest

####### **Correspondence:** Judit Borbely (borbely.judit@dent.semmelweis-univ.hu)

**Abstract**

Background. Agenesis of teeth is one of the most common of human developmental anomalies. Oligodontia is the congenital agenesis of 6 or more permanent teeth.Multidisciplinary treatment approach calls for pre-prosthetic orthodontic space opening for proper positioning of the missing teeth and correction of inter-maxillary relations.High-quality CAD/CAM materials processed by digital workflow offer predictable and reliable treatment options for surgical and prosthetic phases of therapy.

Methods. A 23-year old girl presented with oligodontia and no stable occlusion in premolar, molar region. Two years of orthodontic treatment created space for implant placement but stable occlusion could not be achieved. Diagnostic wax-up was made on casts mounted on articulator (Kavo Protar 5B, Kavo Dental Gmbh, Germany) programmed to individual patient parameters.Optical impression (TRIOS 3, 3Shape, Denmark) was taken as prepreparation scan to record occlusal morphology and contour of crowns. Mock-up served as a guide to follow a minimal invasive preparation approach. Prepared teeth scan was aligned under prepreparation scan and fit to occlusal morphology. Following adhesive cementation of milled hybridceramic occlusal veneers (Vita Enamic, Vita Zahnfabrik, Germany) and stabilizing occlusion digital workflow was used for fixed implant rehabilitation.

Results. A prosthetic-driven backward implant planning and guided implant placement (AstraTech EV Implant System, diameter 3 mm, DentsplySirona Implants, Sweden)(SMART Guide, dicomLAB, Hungary) was performed.  The protocol required conventional impression and cone beam computed tomography (CBCT), the superimposition of dental-gingival information on bone anatomy, surgical planning, 3D-printed teeth-supported surgical templates, and implant placement. After 3 months optical impression was carried out to detect 3D implant position to digitally design temporary crowns(Vita CadTemp, Vita Zahnfabrik, Germany) and soft tissue conditioning was done slightly by flow resin composite. After creating harmonious supraimplant emergence profile intraoral scan was taken and customized titanium-nitride abutments and milled zirconia cut-back crowns (Atlantis, Dentsply Sirona Implants, Sweden) were delivered. Model manufacturing was performed using a 3D printer and screw retained veneered ceramic crowns attached to implants.

Conclusions. This article describes a conventional protocol for creating a mock up to preview esthetics and create proper occlusion using individually programmed articulator and digital workflow of milled occlusal veneers and implant supported crowns for predictable esthetic and reliable functional result.

Written, informed consent for publication was obtained from the patient [or parent/guardian for patients under 16]

#### P30 CAD/CAM Chairside Monolithic Restoration in Esthetic Region with Minimally Invasive Preparation

##### Mariann Danko^1^, Judit Borbely^1^, Zoltan Imre-Kovacs^2^

###### **Correspondence:** Mariann Danko (dankomariann@upcmail.hu)

####### ^1^Semmelweis University, Faculty of Dentistry Department of Prosthodontics - Hungary – Budapest; ^2^Semmelweis University, Faculty of Dentistry, Department of General Dental Preclinical Practice - Hungary - Budapest

**Abstract**

Background. During the development of CAD/CAM technology in dentistry, numerous new ceramic material were introduced for monolithic restorations. Improved optical features of the materials make it possible to produce monolithic restorations also in esthetic region. Reduced wall thickness compared to veneered restorations allows minimally invasive preparation. Chairside design and production unnecessiates 3D printed model. The aim of the study was to describe the chairside CAD/CAM method of making a monolithic crown in the esthetic region with minimally invasive preparation and to compare esthetic features and wall thickness of different material selection: monolithic zirconia, hybridceramic and glass-ceramic.

Methods. A 17-year old girl presented with the need of an esthetic solution for her microdont maxillary central incisor after orthodontic treatment. A minimally invasive treatment concept was used with 0,6 mm reduction. After the rounded shoulder preparation an intraoral scan was taken by Trios POD Wireless scanner. (TRIOS, 3Shape, Denmark) Monolithic crown was designed in 3Shape Design Studio chairside software with the mirroring technique of the contralateral first incisor. Because of the limited space, materials were selected which can be produced with 0,8 mm wall thickness:

1. hybrid ceramic (VITA Enamic HT Multilayer)
2. zirconia-reinforced lithium silicate ceramic (VITA Suprinity PC HT)
3. high-translucency zirconia (VITA YZ HT Color)
4. medium-transucency zirconia (Ivoclar IPS e.max ZirCAD MT Multilayer)
5. low-translucency zirconia (Ivoclar IPS e.max ZirCAD LT)

During the production, the instructions of the manufacturers and clinically proven facts according to the minimum wall thickness were followed. Prosthodontists were asked to compare the crowns using Pink Esthetic Score and White Esthetic Score (PES/WES).

Result. On the basis of the prosthodontists’ comparision and patient choice, the zirconia-reinforced lithium silicate glass ceramic crown had been cemented with adhesive technique.

Conclusions. According to this study it seems that the optical features of the monolithic zirconia and hybrid ceramic restorations in the anterior region are not satisfying yet. Only zirconia and hybrid ceramics can be used in minimally invasive case of 0,8 mm according to the instructions of the manufacturers. Glass-ceramic is not indicated under 1,2 mm circumferential and 1,5 incisal wall thickness. However clinical experiences proved, that with adhesive cementation of a glass-ceramic crown, the reduction is allowed, so the most esthetic zirconia-reinforced lithium silicate ceramic could be cemented.

Written, informed consent for publication was obtained from the patient [or parent/guardian for patients under 16]

#### P31 Digital mockup: evaluation of masticatory muscles function. An observational study

##### Francesca Cattoni, Atanaz Darvizeh, Vincenzo Sanci, Simona Tecco

###### University Vita-Salute San Raffaele, IR.C.C.S. Ospedale San Raffaele - Italy - Milano

####### **Correspondence:** Simona Tecco (simtecc@gmail.com)

**Abstract**

Background. Recently, the roles of occlusion and masticatory muscles have become fundamental in dental rehabilitations. The mockup is used to pre-visualize the final rehabilitations and it can be utilised to test both esthetic and functional patterns before realising the final treatment. The aim of this observational study was to investigate the impact on masticatory muscles by means of measuring electromyographic activity and making a comparison between computer-aided designed/manufactured mockup (digital mockup) and traditional mockup.

Methods. The sample included 22 adult patients, aged 20 to 55 years who were about to receive a prosthetic rehabilitation and did not have any signs of periodontal or systemic disease. A digital or traditional mockup was made for each of the patients. Electromyographic activity of masticatory muscles were evaluated before initiating the treatment (T0), after the mockup insertion (T1), and after treatment (T2) by using the occlusal contact analyzer software called Teethan (Teethan s.p.a., Garbate Milanese, Milan, Italy).

Results. Analysis of parameters at different times for both groups revealed significant variations for most of parameters. The analysis of traditional group revealed a significant variation in POC AT and IMP at “T0 vs. T1” and “T0 vs. T2”, BAR at “T0 vs. T2” and TOT at all 3 time comparisons. In digital group, POC MM and TORS at “T1 vs. T2” and “T0 vs. T2”, BAR at “T0 vs. T1” and “T0 vs. T2” and TOT at all 3 time comparisons showed a significant difference. The comparison between the two groups (traditional and digital) at different time gaps revealed that at ∆1 (T1-T0) only POC AT and IMP showed significant differences and no other significant variation was observed between the two groups at ∆2 (T2-T1) and ∆3 (T2-T0). It concludes that traditional and digital methods generally have nonsignificant differences.

Conclusions. Useful reduction in working time and higher degree of precision in fabrication process of mockup can be obtained by applying the digital technique. Therefore new technological advancement can greatly assist the operator, with a substantially valuable impact. But for ensuring the final restoration of a muscular symmetric function both traditional and digital methods can still be considered to be effective and applicable procedures.

### Science

#### P32 Eagle syndrome: a rare case of calcified ligament with an articulated structure inside

##### Simona Tecco (simtecc@gmail.com)

###### San Raffaele University, Milan, Italy

**Abstract**

Background. Eagle syndrome, or calcification of the stylohyoid ligament, is a rare condition characterized by a mineralization of Mineralization of the stylohyoid ligament occurs infrequently and is usually only an incidental finding noted on routine radiographic examination. Eagle syndrome is diagnosed only when the facial or neck pain originates from a stylohyoid ligament that is calcified. This clinical presentation was identified in 1937 by W.W. Eagle. From a radiographic perspective, Eagle syndrome may be a common incidental finding on routine panoramic radiographs, although only a very small percentage of patients with calcified stylohyoid ligaments present with clinically significant symptoms. Currently, the use of computed tomography (CT) or cone beam CT (CBCT) scanning, with or without three-dimensional reconstruction, is essential to establish an Eagle syndrome diagnosis and to appropriately plan individual patient management.

Methods. This paper reports a case of Eagle syndrome individuated with a CBCT 3D reconstruction, and reviews the literature regarding management options for Eagle syndrome. A case-report of an adult Caucasian patient is reported. The patient has referred to a private practice office in Milan, due to a Temporomandibular joint disorder with neck pain. A complete examination with also a CBCT scanning revealed the presence of an articulated calcified stylohyoid ligament, very particular due to the particular articulation, similar to a knee useful to maintain a mobility of the ligament.

Results. Eagle syndrome may be appropriately diagnosed via a detailed history, physical examination, and radiological evaluation. CBCT and 3D digital reconstructions represent the main diagnostic tool.

Conclusions. This disease process can be confused with many other conditions that must be excluded in the differential diagnosis, mostly through 3D digital reconstructions, to allow appropriate prompt patient management.

### Clinics

#### P33 Digital Orthodontics: Technology in Modern Practice

##### Alessandro Nota, Darvizeh Atanaz, Valter Firbo, Simona Tecco

###### University Vita- Salute San Raffaele, Milan Italy

####### **Correspondence:** Alessandro Nota (dr.alessandro.nota@gmail.com)

**Abstract**

Background. The aim of this study is to describe the integration of technology with clinical orthodontics. New digital technologies appear essential to the success of any orthodontic practice seeking to increase efficiency and communication. The aim of the present study is to describe a practical digital workflow in clinical orthodontics.

Methods. A useful workflow to integrate digital impression, model storage, orthodontic treatment planning, and customizing the orthodontic appliances with the support of a dental laboratory is described. Advantages are exposed, and limits are discussed, with a particular focus on the clinical point of view.

Results. Digital technology could enhance the ability of the orthodontist to diagnose and treatment plan, using high resolution graphics and model analysis measurements. It also could allow the orthodontist to easily interact with the laboratory, improving the final satisfaction of the patients.

Conclusions. A whole digital workflow appears particularly useful in orthodontics also to reduce estimates costs for manufacturing of oral appliances.

#### P34 Effectiveness of Shade Measurement Using Intraoral Scanner Compared with Digital Spectrophotometer and Visual Shade Assessment

##### Ivett Roth (yvettroth@gmail.com)

###### Semmelweiss University, Budapest, Hungary

**Abstract**

Background. In digital shade measurement digital spectrophotometer is used as a gold standard. New intraoral scanner system was developed with a tool for teeth shade measurement, but there is less information about its efficiency. The aim of this study was to evaluate shade measurement function of a digital scanning system in relation to visual shade determination and digital spectrophotometric device.

Methods. 10 dental students from Semmelweis University took digital impressions and measured tooth shade for 10 different patients using 3Shape Trios intraoral scanner (TR). Students also selected shade using 2 visual methods: Vita A1-D4 (VC) and Vita Linearguide 3D-Master (LG) shade guides and an instrumental method: Vita Easyshade (ES). The study was ethically approved by the Semmelweis University Institutional Review Board (SE-TUKEB 61/2016). The inclusion criteria of patients were full dentition and no prosthetic restorative treatment. Students had no previous experience in intraoral scanning and tooth color matching, they attended an education course: lecture and training on intraoral scanning and shade matching methods with shade tabs and digital devices. The students matched the color of 3 teeth for each patient: 11 – cervical, central, incisal, 14 – central, 16 – central. Time spent on shade matching were recorded. For each tooth there was a VC shade- tab, an LG shade- tab and LG-shade tab according to ES measurement. Four selected shade tabs were presented to the patient, the student and an experienced dentist to select the best match. The results were recorded and evaluation of percentage was calculated.

Results. The percentages of the selected best matching shade tabs out of the 4 methods: LG: 35,08%, ES: 26,58%, TR: 21,64%, VC: 16,7%. Time spent on shade matching by the 4 different shade matching methods: ES: 14,12 sec, TR:40,06 sec, VC: 52,42 sec, LG: 70,47 sec.

Conclusions. Intraoral scanner (TR) was acceptable for shade measurement. In case of digital impression taking using another device for shade matching wasn’t necessary, color results were showed automatically. The fastest shade matching procedure was digital spectrophotometer (ES) followed by intraoral scanner. Most “best fit shade tabs” were selected with visual method (LG). Shade selection with visual method took the most time.

#### P35 A sport injury of the left mandibular angle in a young adult allows to discover a morphological alteration of the other condyle. A case report

##### Atanaz Darvizeh, Alessandro Nota, Simona Tecco

###### University Vita- Salute San Raffaele, Milan, Italy

####### **Correspondence:** Atanaz Darvizeh (atanazdarvizeh@gmail.com)

**Abstract**

Background. The purpose of this work is to present a clinical case in which digital image analysis has allowed a more accurate diagnosis in the context of Temporomandibular disorders (TMDs)

Methods. The clinical case of a patient who presented himself to our observation due to a sports trauma (a calcium) directed to the left mandibular angle, is described. Immediately after trauma, the patient was hospitalized in a maxillofacial unit of another structure, where he was subjected to a two-jaws block in protruded mandibular position for a period of some weeks, after which he has been resigned. After some months, he has turned to our structure for signs and symptoms of TMD, and malocclusion. The patient was prescribed an RMI of the Temporo-mandibular joints, a CBCT and routine investigations.

Results. The analysis of digital records allowed the diagnosis of a morphological anomaly of the right condyle, which had not been detected previously, since at the maxillofacial department only an analysis of the left side (involved in the trauma) has been performed

Conclusions. The present clinical case suggests that, in contrast to the current clinical guidelines, in case of trauma involving the jaw, mostly the Temporo-mandibular joints, a gnathological evaluation by a specialist dentist should always be considered.

#### P36 Guided, socket shield, immediate loaded implantation, case report

##### Lukasz Zadrozny, Leopold Wagner

###### Medical University of Warsaw - Poland

####### **Correspondence:** Lukasz Zadrozny (lukasz.zadrozny@gmail.com)

**Abstract**

Background. Proper implant positioning in the post-extraction socket is crucial for the function, durability and aesthetics of the future prosthetics. In the esthetic zone even more challenging and very difficult is to maintain the shape and structure of the tissues surrounding the tooth after its removal. Literature presents many techniques aimed at preserving tissues after tooth extraction in the best possible condition. One of them, described in 2010 by M. Hurzeller, whom proposed the retention of the buccal root segment after extraction- Socket-Shield technique. Other authors analyse it histologically and have presented many clinical cases. Furthermore, socket shield technique was classified as one of partial extraction techniques which is highly promising and may significantly influence management of the failing dentition, changing a concept from extract and augment to salvaging the patient’s own tissues where possible.

Methods. This clinical report presents digitally guided approach to perform partial extraction therapy where the tooth has to be replaced by an implant immediately. A 38-year-old female patient presented fracture of clinical crown in tooth 21. Clinical examination revealed total mobility of composite crown from palatal site. Because of direction and depth of crown fracture the tooth was classified to be extracted. Digital model of patient dentition was prepared as the stl file and superimposed over dicom files from CBCT examination. Implant position and surgical guide were digitally planed in DDS-Pro software (DDS-Pro). Temporary crown was designed in Exocad (Exocad Gmbh) software and milled in PMMA. After preparing of buccal root segment the osteotomy for 4,0x11,5 implant (Hiossen TSIII NH, Osstem Implants) was performed though 3D printed sleeveless guide with OneGuide Kit (Osstem Implants). Implant was placed palatal to the shield with initial stability 35 Ncm. PMMA crown was bonded to temporary abutment and attached to an implant with torque of 20 Ncm.

Results. Postopperative CBCT revealed proper implant positioning and maintaining a buccal root shield.

Conclusion. The modern dental care philosophy aims to preserve dental tissue as much as possible, even when tooth extraction is required. Very important are also time of treatment and addressing aesthetic and functional expectations of the patient. Presented case required a thorough planning phase but offered a possibility of not loosing a tooth from aesthetic point of patient’s view. Considering perfect implant positioning and maintaining the best condition of hard and soft tissues, presented in this study technique, combining PET with guided implantation, could be a valuable tool to restore teeth especially in the aesthetic region.

Written, informed consent for publication was obtained from the patient [or parent/guardian for patients under 16]

### Science

#### P37 Graphical principle of STL superimposition: approbation of complex approach in peri-implant results evaluation

##### Goncharuk-Khomyn Myroslav (myroslav.goncharuk-khomyn@uzhnu.edu.ua)

###### DDS, MMSc, PhD-candidate, Head of the Scientific and Research Centre of Forensic Dentistry, Teaching Assistant at Uzhhorod National University (Ukraine)

**Abstract**

Background. The objective of research was to approbate integrated approach for evaluation the results of dental implant treatment using customized image analysis algorithm of CBCT scans.

Methods. Primary stage of the research was dedicated to the formation of a study sample, consisted of a set of 156 CBCT data pairs (dicom-files, voxel size 100 μm, isotropic) of 78 patients who had undergone dental implantation at the University Dental Clinic. Periimplant bone changes evaluation was following originally developed algorithm which included, segmentation of the area of interest from the CBCT data set with the isolation of the implant installation region, transformation of the formatted image of region of interestI from the *.dcm format to the *.stl format, superimpose of obtained stl-files from the data sets, calculation of the absolute bone volume loss around dental implant due to the amount of non-matched image parts. Evaluation of bone parameters conducted in the adapted software ImageJ with additional plug-in BoneJ (the Wellcome Trust). Implant stability was measured with Ostell Mentor (Ostell). Statistical analysis of numerical data performed using software Microsoft Excel software (Microsoft Office, 2016).

Results. Method of STL-superimposition helped to calculate absolute volumetric parameter of radiused bone loss at periimplant region. Such average values for the implants installed in the distal part of maxilla were 3,547 cubed mm., around maxillary frontal implants its volume reached 3,118 cubed mm., mean bone loss around mandibular distal implants was 2,614 cubed mm., and mandibular frontal implants lost 2,456 cubed mm. of circular bone volume. Statistically tested correlation value between mean vertical bone loss and radiused volumetric bone reduction reached r=0,784 (p≤0,05). Also specific regressions between parameters of bone loss and prosthetic designs were found. In addition correlation level were estimated between ISQ values, volumetric bone loss measured by the superimposition principle and marginal bone loss measured by the sagittal slices of CBCT.

Conclusions. The proposed approach ensures possibilities for objectification not only geometrical, but also qualitative changes of bone at peri-implant region and takes into account the impact of functional stability of titanium infra-structures to prognose the success of treatment. The possibility of considering customized values of volumetric peri-implant bone loss instead of simple geometrical bone height reduction value, allows to individualize treatment algorithm by adapting different prosthetic designs due to the patient rehabilitation group with different trends of bone loss.

### Clinics

#### P38 CAD/CAM Monolithic Restoration in Molar Region without Occlusal Adjusment

##### Zoltán Imre Kovács^1^, Judit Borbely^2^, Peter Schmidt^2^, Peter Hermann^2^

###### ^1^Semmelweis University, Faculty of Dentistry, Department of General Dental Preclinical Practice - Hungary – Budapest; ^2^Semmelweis University, Faculty of Dentistry, Department of Prosthodontics - Hungary - Budapest

####### **Correspondence:** Zoltán Imre Kovács (dr.csonti@gmail.com)

**Abstract**

Background. Development of digital technology in dentistry gives the opporunity to produce CAD/CAM temporary and definitvie monolithic restorations. A usual problem with monolithic restorations is the occlusal correction after syntering procedures. During dental treatment, usually a temporary restoration is needed, which fits the preparation margin and the occlusion is adjusted in the patient mouth. If the morfology of this restoration is perfect, the need of making an absolutly alike definitve restoration appears. Laboratory design software makes it possible to use shapes recorded in prepreparation scan during the comptuter aided design. The aim of this study was to compare the adjusted occlusal contact of the temporary restoration to the monolithic definitive restoration’s, which shape is copied from the temporary in the design software.

Methods. A 36 year old woman presented with the need of a treatment for her lost left mandibular first molar. The treatment plan was a 3 unit monolithic zirconia bridge. The preparation was made with 1-1,5 mm reduction and rounded shoulder finishing line. An intraoral scan was taken by Trios POD Wireless scanner. (TRIOS, 3Shape, Denmark). A 3 unit temporary PMMA bridge (Yamahachi) was designed in 3Shape Dental Designer software. After milling, the occlusion was adjusted in the mouth with the help of a T-scan device (Tekscan, U.S.). A prepreparation intaoral scan was taken from the temporary bridge, which was copied in the design software for the definitive monolithic zirconia restoration. (GLI Bruxzir shaded, Glidewell Dental, U.S).

Results. The measured occlusal contacts with T-scan of the definitive restoration did not show significant difference from the temporary restorations.

Conclusions. Using the recorded shape of the temporary bridge as a prepreparation scan, the production of the monolithic zirconia bridge was possible. The patient felt the zirconia bridge as comfortable as the PMMA. With this method, the occlusal correction and repolishing of the definitive restoration was not needed.

Written, informed consent for publication was obtained from the patient [or parent/guardian for patients under 16]

#### P39 Complete digital workflow and immediate functional loading of implant-supported monolithic glass ceramic crowns

##### Justinas Pletkus^1^, Adomas Auškalnis^2^, Marius Kubilius^3^, Jotautas Kaktys^4^, Ieva Gendvilienė^5^, Rokas Borusevičius^6^, Agnė Gečiauskaitė^7^, Vygandas Rutkūnas^8^

###### ^1^Resident at Department of Prosthodontics, Institute of Odontology, Faculty of Medicine, Vilnius University, Lithuania; Private practice; ^2^PhD, lector at Department of Dental and Oral Pathology, Faculty of Odonthology, Lithuanian University of Health Sciences; Private practice ("Restauracines odontologijos centras", Kaunas, Lithuania); ^3^PhD, DDS, Private practice ("Restauracines odontologijos centras", Kaunas, Lithuania); ^4^Private practice ("Restauracines odontologijos centras", Kaunas, Lithuania); ^5^Department of Prosthodontics, Institute of Odontology, Faculty of Medicine, Vilnius University, Lithuania; Private practice (“Prodentum”, Vilnius, Lithuania); ^6^Rokas Borusevičius, PHD student at Department of Periodonthology, Institute of Odontology, Faculty of Medicine, Vilnius University, Private practice; ^7^PhD student, Department of Prosthodontics, Institute of Odontology, Faculty of Medicine, Vilnius University, Lithuania; Private practice (“Prodentum”, Vilnius, Lithuania); ^8^Associate Professor, Department of Prosthodontics, Institute of Odontology, Faculty of Medicine, Vilnius University, Lithuania; Private practice (“Prodentum”, Vilnius, Lithuania).

####### **Correspondence:** Justinas Pletkus (justinas.pletkus@gmail.com)

**Abstract**

Background. Survival and success rates of immediate implant loading may vary widely according to literature. Advances in digital technologies create an opportunity for fast fabrication and delivery of final restorations. There is a very limited data on clinical outcomes of immediate loading of implants with final full-contour ceramic restorations produced using completely digital workflow. The aim of this study was to evaluate surgical and prosthetic aspects of immediate implant loading with glass ceramic screw-retained single crowns that could be associated with biologic and prosthetic outcomes.

Methods. Nineteen subjects, who required single tooth implant supported crown in the posterior region of the mandible received one or more implant (patients with bad or parafunctional habits were excluded). In total 22 implants were placed. Primary stability measured and recorded in Ncm and ISQ values. Cerec® (Densply Sirona) implant scan post and scanbody was attached to the implant. Omnicam® (Densply Sirona) intraoral scanner (IOS) (CEREC AC software 4.3) was used to take the digital impressions and bite registrations. Within 24hours functional glass ceramic (N!CE, Straumann) crowns were delivered (Fig. 1 and Fig. 2). For every crown occlusal and interproximal contacts were adjusted and recorded. Quality of contact points were measured with shimstock occlusal foil (Hanel, Coltene). Restorations were followed-up for 1, 3, 6 months.

Results. 1 implant failed and was removed after 4 weeks. The rest implants and crowns were in function with no technical or biological complications after 6 months of use (survival rate of our study was 95,5%). Statistically significant effect on MBL was not found considering factors such as implant size, time after extraction, bone type, soft tissue thickness, and primary stability measured in Ncm and ISQ values. Mean MBL mesially was 0.3mm (SD 0.42) and distally – 0.4mm (SD 0.66). These findings are similar to those in other studies reporting MBL on delayed or immediate loading. One distal and one mesial proximal contacts were missing during 6 months check-up, similarly as during insertion of the crowns - 1 and 2, accordingly.

Conclusions. Fully digital workflow without 3D printed model could be successfully employed for immediate functional loading with a single-unit implant-supported crowns fabricated from glass ceramics. When patients are carefully selected, it can provide clinically acceptable biologic and prosthetic outcomes in a follow-up period of 6 months. The protocol should be further evaluated with longer observational periods.

#### P40 Unconventional Implant Placement Through Impacted Maxillary Canine – A Case Report

##### Ivona Bjenjaš^1^, Nenad Milinković^2^, Đorđe Pejanović^3^, Tamara Ristić^3^

###### ^1^Dental clinic “BGD Osmeh” Francuska 25 11158 Belgrade; ^2^Dental SM D.O.O. Bulevar Oslobođenja 152 11040 Belgrade; ^3^Faculty of Dentistry Pancevo Žarka Zrenjanina 179, 26000 Pancevo; Faculty of Dentistry Pancevo Žarka Zrenjanina 179, 26000 Pancevo

####### **Correspondence:** Ivona Bjenjaš (ivonabjenjas@gmail.com)

**Abstract:**

Background. Female patient 66 years old needed dental therapy of the upper right partial edentulism of maxilla. Old PMF fixed partial denture was loosened and the extraction of the tooth 12 was indicated. Tooth 15 was indicated for the root treatment and post. Region 14 and 13 were indicated for implant therapy.

Methods. Dental status was taken. To reduce the patient x-ray exposure following ALARA recommendation only small FOV on CBCT was done. The impacted right upper canine was discovered. Patient knew about it, but it was completely asymptomatic for years and no other surrounding pathology couldn’t be seen on CBCT. Extraction of the impacted tooth was avoided because of expected massive bone loss and possible complications. The tooth 12 was extracted. There was no buccal plate after extraction, so this site was excluded for immediate implant placement. Two C-Tech implants were placed through the impacted tooth in region 13 (EL 3,5x11) and 14 (EL 3,5x9). Primary stability was achieved on 60N/cm. The wound was sutured with 4,0 Nylon. Control CBCT was taken, expected positions of implants were achieved. Patient was prescribed with antibiotic therapy Sinacillin 0.5g 3x1 for seven days and Ibuprofen 0.4 if needed.

Results. No postoperative pain, swelling or bleeding was reported by the patient. After seven days sutures were removed, the wound healing was uneventful. Tooth 15 was treated and CoCrMo post was made. After four months implants were uncovered without any difficulties and healing caps were placed.

Conclusions. There are only a few case reports done with placing implants into impacted teeth. From our experience protocol deviation is justified in situations when expected bone loss is compromising implant therapy afterward. There should be more studies confirming the safety of such procedures.

Written, informed consent for publication was obtained from the patient [or parent/guardian for patients under 16]

#### P41 Treatment of Ameloblastoma in posterior mandible with resection, reconstruction by free fibula graft and dental rehabilitation of conventional dental implants

##### Ashwini Bhalerao^1,2^ (ashwinib1170@gmail.com)

###### ^1^Private Practice, Mumbai, India; ^2^Consultant oral and maxillofacial surgeon, Criticare group of hospitals, Mumbai, India

**Abstract**

Background. Ameloblastoma is a benign, locally infiltrative epithelial odontogenic tumour with cortical expansion and a high local recurrence rate. Clinically; the long standing lesions are characterized by looseness of teeth, root resorption and usually combined with unerupted tooth. Tumour cells have a great tendency to invade the surrounding healthy tissue which is considered to be the essential step in tumour progression. In the mandible majority of ameloblastomas are found in the molar ramus region. There are 7 types of ameloblastomas, follicular ameloblastoma having the highest rate of recurrence, therefore these cases are treated by surgical excision and reconstruction by iliac crest or free fibula graft. The challenge is in dental rehabilitation of such patients, aim is to rehabilitate the dentition not with removable but with dental implants restoring form and function.

Methods. A 28 years old male came with complaint of pain, swelling and discharge from right retromolar region since 3 weeks’ revealed presence of a huge cystic lesion in the right ramus of mandible which had pushed the third molar superiorly. What was first thought to be a routine dentigerous cyst turned out to be a follicular ameloblastoma, confirmed by biopsy. Under general anaesthesia, the patient was operated for complete enucleation of the tumour. The Mandible was partially resected, simultaneously a free fibula graft was harvested and fixed in place with a reconstruction plate. The patient was placed on intravenous antibiotics and painkillers, after 8 days, he was discharged. Liquid diet was advised for 3 weeks post-surgery. After 10 months, the soft tissue healing was complete, CT scan showed a well-integrated graft. The cortico cancellous component was deemed to be satisfactory and dental implants placed by a flapless procedure as we did not want to disturb the mucosal attachment to the graft. 8 weeks later, they were loaded with screw retained prosthesis to prevent cement leaching through the mucosa till the fibula. Occlusion was checked with a T scan to ensure equal distribution.

Results. Satisfactory dental rehabilitation was achieved by placing dental implants in the optimal stage when the cortico cancellous proportion of fibula graft was favourable for achieving primary stability.

In the follow up of two years, we saw good remodelling of fibula under favourable load Conclusions. Patients with resected mandible can be given fixed prosthetic options, provided the planning and placing of dental implants is done in a calculated phased manner and the patient is counselled on the need of strict oral hygiene measures in the absence of attached gingiva. Giving such patients a removable denture or obturator works against them as food collection, non-loading, leads to resorption of graft and facial asymmetry with loss of function.

Written, informed consent for publication was obtained from the patient [or parent/guardian for patients under 16]

### Science

#### P42 The Impact of an Augmented Reality Application on Perception and Anxiety Level in Students during Administration of First Local Anesthetic Injection

##### Rasa Mladenovic^1^, Leandro Pereira^2^, Kristina Mladenovic^3^

###### ^1^Faculty of Medicine, University of Pristina, Kosovska Mitrovica, Serbia; ^2^Blantus Endodontic Center, Campinas, Brazil; ^3^Faculty of Medical Sciences, University of Kragujevac, Serbia

####### **Correspondence:** Rasa Mladenovic (rasa.mladenovic@med.pr.ac.rs)

**Abstract**

Background. Although local anesthesia (LA) is an imperative of modern dentistry, many dental students feel insufficiently prepared for administration of their first injection in a clinical setting. Augmented Reality (AR) is a technology that superimposes a computer-generated image on a user's view of the real world, thus providing a composite view. Visualization and interaction are fundamental components of AR technology. The objective of this study was to examine the impact of an AR application in students administering LA for the first time.

Methods. Students were categorized into two groups. In addition to the theoretical and practical training, the students of the experimental group used the AR simulator. The trigger for acute stress was LA administration. The concentration of salivary cortisol was measured before and after LA. Patients were children who had indications for anesthesia. Mann-Whitney test, t-test and Wilcoxon test were used to test statistical hypotheses.

Results. The level of cortisol after anesthesia was statistically significant in all subjects, but there was no statistically significant difference between the groups. The time required for the LA administration was significantly longer in the control group (p = 0.014).

Conclusions. Given that blood sampling is a stressful process that could add up to the anxiety level, in the study we measured the concentration of salivary cortisol. We opted for afternoon measurements when the levels of salivary cortisol are considered to be stable according to the circadian rhythm. Increased cortisol concentration in all students after LA supports the opinion of many clinicians that LA administration is one of the more stressful aspects of clinical practice. Having the syringe in the patient’s line of sight and performing lengthy treatments may result in unsatisfactory analgesia, specifically in children. Using the AR, the anatomical structures in the oral cavity are easy and convenient to use and give a good introduction to clinical anesthesia due to user interaction, as demonstrated in our study - students who received AR training had significantly less time for the anesthetic procedure. This form of learning provides an innovative and interactive way of presenting material, and therefore should be used in addition to conventional learning or as a self-improvement tool that can result in a positive response and student motivation. AR application can influence better understanding of anatomy structure and the correct and faster technique for administering local anesthesia, but cannot reduce acute stress.

### Clinics

#### P43 Influence of malocclusion and cranio-cervical disfunction in facial paralysis

##### Diego Tatis^1,2^ (dtatis@orthokinetic.com)

###### ^1^Isaac Newton Universitiy Costa Rica; ^2^Orthokinetic Training Center – Colombia

**Abstract**

Background. To present and to explain the influence of dental occlusion, the cranio-masticatory system and the cranio-cervical system in the etiology and prognosis in the therapeutic management of peripheral paralysis of the facial nerve. In this work, the paralysis of the VII cranial nerve refers to an acute unilateral peripheral facial paresis, mono systomatic, until now, in most cases unknown.

Methods. The material of this poster includes a literature review and two clinical cases of perispheric facial nerve palsy from the group of cases studied and treated by the author over a period of 20 years. Patients previously evaluated and diagnosed by neurosurgery with facial paralysis or Bell's palsy. A common clinical pattern was observed in the treated patients: skeletal class II, facial asymmetry, temporo-mandibular dysfunction and cranio-cervical dysfunction. A standard test was performed at the first consultation, which included a detailed description of the degree and location of the tests. of paresis, taste, extra-oral clinical examination and intra-oral clinical examination, electromyographic examination, MRI and audio-spectrometric analysis of temporomandibular joints. The immediate suspension of any type of medication indicated previously for the treatment of the pathology is ordered. OrthokineticTM splints of Neuro Muscular and Skull-cervical Articular Repositioning Splints (Patent No. 978627) are adapted and controlled with T-Scan Analysis and follow-up once every two days, second control at week and third at two weeks and then once a month. Complementary phisical therapy was used.

Results. The clinical examination revealed 100% unilateral complete paralysis. The follow-up showed in 100% of the patients the function was partially restored on the second day and total at 6 days. In 100% of the patients the function of the normal mimicry was obtained.The sequels of dysfunction were 0% for the total of the patients

Conclusions. Cranio-masticatory dysfunction could have an influence on skeletal mechanics and neuro-muscular mechanics in the anatomical course of the facial nerve, which could lead to its dysfunction, with the consequent paralysis of the muscles of the affected side.The use of prednisone poses a great ethical problem because there is no evidence of its efficacy and the euphoric side effect induces a false sense of benefit in patients.Dental occlusion is directly and intimately related with the temporo-mandibular and cranio-cervical balance. Occlusal imbalances could influence neuro-muscular imbalance, temporo-mandibular imbalance and cranio-cervical imbalance, which would affect the normal function of any cranial nerve involved, including the facial nerve or VII cranial nerve.

#### P44 Guided implant placement in patient with Multiple Idiopathic Cervical Resorption

##### Lukasz Zadrozny, Katarzyna Brus-Sawczuk

###### Medical University of Warsaw - Poland

####### **Correspondence:** Lukasz Zadrozny (lukasz.zadrozny@gmail.com)

**Abstract**

Background. Multiple Idiopathic Cervical Resorption is very a rare disease and proceed asimptiomatic until teeth mobility occurs or accidental X-ray reveales different degree of cervical resorption in more than one tooth. Infected teeth stays vital with proper pulp reactions. MICR may run and develop in different time on another teeth even if previously founded illness teeth were treated or extracted. Potential reasons for this disease developent are trauma or injury or systemic diseases such as hyperparathyroidism.

Methods. A 41-year-old female patient with good general health complained on increased mobility of front teeth in the mandible. Clinically teeth 32-42 has 3^rd^ grate of mobility. Patient had a face trauma about 30 years ago. OPG X-Ray revealed severe cervical resorption in teeth 33-43 and 15-11, 21 has RCT done many years ago. CBCT was taken for more accurate diagnostics and additional blood test were done. Mandible treatment plan based on virtual planning with CBCTs DICOM data and an optical scans STLs files of both dental arches (DentiqGuide) and preparing temporary bridge (Exocad) obtains extracting of teeth 33-43 and guided immediate implants placement in positions 33, 31, 43 and immediate loading. In local anesthesia teeth 33-43 were sectioned and extracted. Sleeveless 3D printed guide with OneGuide Kit (Osstem Implant) were used to place implants in positions corresponding with virtual planning to be restore immediate by already milled from PMMA six units temporary bridge. ISQ values for each implant was over 75 what provides condition for immediate loading. Bridge was positioned in occlusion with positioning splint and bonding to abutments. After unscrewing, bridge was polished and screwed back, access holes were closed with composite.

Results. Teeth supported 3D printed guide provides 3 implants placement with accuracy allowing for fixing and screw retaining of six points unit bridge, in proper occlusion.

Conclusion. Virtual, backward planning of complex cases may offer possibilities for an esthetic restoration of implant supported fixed prosthesis. In the presented case of MICR, treatment plan includes potential development of resorption on remaining teeth. Basing on that fact long time temporary reconstruction was attached to the implants through multiunits. Implants positioned in sites 33, 31 and 43 and multiunit abutments creates open condition to exchange bridge to more expand if potential further extractions would be required and offers even full arch reconstruction with only two additional implants in premolar or molar sites if the initial disease would still develop.

Written, informed consent for publication was obtained from the patient [or parent/guardian for patients under 16]

#### P45 Potentially mortal complication during guided implant placement in anterior mandible

##### Lukasz Zadrozny, Leopold Wagner

###### Medical University of Warsaw - Poland

####### **Correspondence:** Lukasz Zadrozny (lukasz.zadrozny@gmail.com)

**Abstract**

Background. Frontal mandible is common described as a safe zone for implant procedures. Besides of inferior alveolar nerves and vessels injuries much more dangerous may be hurting of sublingual artery or its branches what can happen if implant drill perforate lingual plate of mandible. Hurting these arteries may provoke severe bleeding to the sublingual space and creation of sublingual hematoma and subsequently obstruct the airways, what can be potentially mortal.

Methods. A 62-year-old female patient presented with hoopless teeth 33-41 in anterior mandible and toothless maxila. Teeth extractions were planed and because of big discrepancy in bone height of front and back segments of mandible, bone reduction in front segment was planned. Two implants with Locator attachments in positions of canines were planned to support future removable prosthesis. Two- pieces bone supported surgical guide was designed and 3D printed. First- lower part fixed to the bone with two anchor screws determines range of planned bone reduction. Second- upper part of guide with attachments to the first and another bone anchor pin provides implants positioning with sleeveless OneGuide Kit (Osstem Implants). After flap opening, teeth were extracted and first part of guide were set on the bone and stabilize with two anchor screws. Bone was reduced according to the guide. Second guide part was then attached to the already fixed part and additionally fixed with third anchor. Osteotomy for implant in position 33 was performed. During preparation of osteotomy in position 43 high bone density was felt. Probing revealed lingual plate bone perforation at the end of osteotomy. No severe bleeding occurred and no distortion of lingual soft tissue were found. Osteotomy was filled with hemostatic sponge (Ferrosan) and medially another osteotomy was prepared freehand. Implants were placed and wound was closed.

Results. Check up examinations and communication with patient didn’t revealed any signs of increasing bleeding and development of intraoperative complication.

Conclusion. Guided surgeries provide new possibilities and improve accuracy of implant procedures. Very important is to not forget about still existing range of inaccuracy especially when tissue or bone supported guides are used. Clinician has to consider that drills for guided surgeries are about 10mm longer than standard and the longer the drill the farer its tip may deflect even with minor guide setting inaccuracy. That may leads to destroy anatomical structures, and it’s extremely important to calculate safety zones even in guided surgeries.

Written, informed consent for publication was obtained from the patient [or parent/guardian for patients under 16]

